# Land use dynamics and their impact on hydrology and water quality of a river catchment: a comprehensive analysis and future scenario

**DOI:** 10.1007/s11356-025-35946-y

**Published:** 2025-01-25

**Authors:** Natnael Shiferaw, Lulit Habte, Mirza Waleed

**Affiliations:** 1 Australian Rivers Institute, School of Environment & Science, Griffith University, Nathan, QLD 4111 Australia; 2 Julius Kruttschnitt Mineral Research Center, Sustainable Minerals Institute, University of Queensland, Indooroopilly, QLD 4068 Australia; 3 Department of Geography, Hong Kong Baptist University, Hong Kong SAR, China

**Keywords:** Land use, Water quantity, Water quality, HSPF, FLUS

## Abstract

Land use changes profoundly affect hydrological processes and water quality at various scales, necessitating a comprehensive understanding of sustainable water resource management. This paper investigates the implications of land use alterations in the Gap-Cheon watershed, analyzing data from 2012 and 2022 and predicting changes up to 2052 using the Future Land Use Simulation (FLUS) model. The study employs the Hydrological Simulation Program-FORTRAN (HSPF) model to assess water quantity and quality dynamics. Seven land use classes were identified, and their evolution was examined, revealing significant shifts in urban, agricultural, grassland, wetland, and forested areas. The model performance across observed data was evaluated using coefficient of determination (*R*^2^), percent bias (PBAIS), and mean absolute error (MAE). Results show the dynamic nature of land use changes, highlighting shifts in urbanization, agriculture, and forested areas. Notably, the study explores the consequences of these changes on water quantity and quality, scrutinizing surface runoff, evapotranspiration, stream flow, and nutrient loads. Urban green spaces emerge as key mitigators, regulating runoff and enhancing water absorption. Forests (vegetation) also play a crucial role in maintaining water balance, while wetlands act as natural filters for flood mitigation and water quality improvement. The findings underscore the importance of informed land use planning, recognizing urban green spaces, forests, and wetlands as integral components for sustainable watershed management. As society navigates environmental challenges, this research contributes to a deeper understanding of the complex interactions between human activities and the natural environment emphasizing the need for nature-based solutions in land use planning for resilient and balanced ecosystems.

**Supplementary Information:**

The online version contains supplementary material available at 10.1007/s11356-025-35946-y.

## Introduction

The changes in land use significantly affect hydrological processes, soil properties, and water quality at local, regional, and global scales (Wan et al. 2014). A variety of land use changes can significantly impact the water cycle, changing water availability and water quality as a result of deforestation, urbanization, agricultural expansion, and construction of impervious surfaces (Namugize et al. 2018). Understanding these effects is crucial for sustainable water resource management and environmental planning. Alterations in land use have been recognized as the primary driving factor propelling environmental changes at the local, regional, and global levels, gaining growing emphasis in assessing the human-induced impacts on the environment (Verburg et al. 2015). Land use changes are the result of dynamic human–environment interactions in processes operating at different spatiotemporal scales (Aquilué et al. 2017; Verburg and Overmars 2009).

Changes in land use directly influence water quantity by altering the processes of evapotranspiration, infiltration, runoff, and groundwater recharge. Deforestation, for instance, reduces the amount of interception and changes soil properties such as conductivity and water-holding capacity, resulting in increased surface runoff and decreased groundwater recharge (Owuor et al. 2016). This can lead to more frequent and severe flooding events and reduced river baseflow during dry periods. Conversely, agricultural practices like irrigation can increase water consumption and minimize streamflow and groundwater levels. Land use changes can also have significant impacts on water quality. As a result of impervious surfaces such as roads and buildings in urban areas, surface runoff carries pollutants into nearby water bodies, including sediments, nutrients, heavy metals, and chemicals (Wilson and Weng 2010). In the same way, agricultural activities, such as the use of fertilizers and pesticides, are capable of contaminating surface and groundwater (Shiferaw et al. 2023). Deforestation can also impact water quality by increasing erosion and sedimentation, degrading aquatic habitats, and impairing water ecosystems.

Recent attention to land use change and future landscape patterns highlights the usefulness of land use models for exploring land use change and management (Ren et al. 2019; Sapena and Ruiz 2015). The FLUS model simulates land use change and future scenarios under the influence of human activities and nature (Chen et al. 2021). The FLUS model is more effective at handling non-linear relationships by avoiding error transmission than traditional cellular automata (CA) based models because it only samples from the most recent period (Chen et al. 2021).

Since hydrology is the driving force behind many watershed processes, hydrological modeling is crucial for watershed management and explains the mechanisms (Albek et al. 2004; Li et al. 2015). HSPF is a comprehensive model developed by the United States Environmental Protection Agency (USEPA) that has operated in conjunction with BASINS (Better Assessment Science Integrating Point and Non-Point Source) to simulate the quality and quantity of water in watersheds of various sizes and complexity levels. It can simulate watershed hydrology under various changes in land use and climate and the hydraulics of dams and reservoirs (Chen et al. 2019; Kim and Choi 2020; Roostaee and Deng 2019; Yazdi et al. 2019).

Despite extensive research on land use changes, gaps remain in understanding the mechanisms of land use changes, especially in de-urbanized areas, and long-term effects on watershed hydrology and water quality. This paper aims to comprehensively analyze the impact of land use changes on hydrological processes and water quality in a watershed where urban areas are vastly reduced. It also aims to investigate the specific alterations over the years and under future scenarios. Moreover, the paper aims to highlight the key drivers of land use change and elucidate their consequences on watershed dynamics.

## Materials and methods

### Study area

**Fig. 1:**
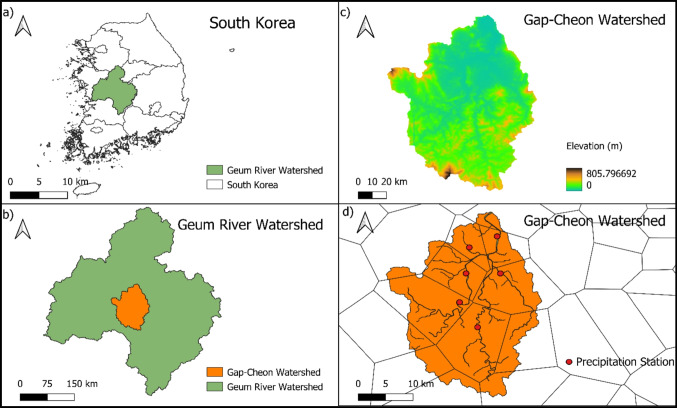
Study area **a** map of South Korea, **b** Geum River watershed, **c** DEM of Gap-Cheon watershed, and **d** Thiessen polygon and precipitation station

The Gap-Cheon watershed, situated in the central-west region of South Korea, covers an area of approximately 636 km^2^, as shown in Fig. 1. With a population nearing 1,470,000, it encompasses Daejeon Metropolitian City, the fifth-largest city in South Korea. Over the past decade, this watershed has seen a noticeable decline in the population. The Gap-Cheon River is one of the major tributaries of the Geum River, and it plays a significant role in the city’s geography and culture. Gap-Cheon River originates from Daedunsan mountain and flows north toward Daejeon before flowing into the Geum River. It provides a water source for various uses, including drinking, irrigation, agriculture, and industrial purposes. The Gap-Cheon watershed has seven land uses: urban land, agricultural land, forest land, grassland, wetland, barren, and water. The watershed has undergone substantial land use changes, with urbanization being the most prominent transformation up to 2010. During this period, urban areas expanded by approximately 7% from 1990 to 2010. In recent years, however, there has been a noticeable shift in this trend, which changes both the water quantity and quality of the watershed.

### Data preparation

Sub-daily weather data such as precipitation, potential evapotranspiration, evaporation, wind speed, temperature, solar radiation, evaporation, dew point, and cloud cover were collected from the Korean National Satabase System (data.kma.go.kr). A 30-m resolution digital elevation model (DEM) was collected from the National Geographic Information Institute (ngii.go.kr). Land use data were collected from the Environmental Spatial Information service (egis.me.go.kr). Flow rates and water quality data were collected from the Water Resources Management Information System (wamis.go.kr).

### Hydrological and water quality model

#### Hydrologic Simulation Program Fortran (HSPF)

In this work, the HSPF model was run from BASINS (Better Assessment Science Integrating Point and Non-Point Source), a GIS-based system that integrates environmental data, analytical tools, and modeling programs. HSPF was first developed as an extension and refinement of the Stanford watershed model (Crawford and Linsley 1966). HSPF is a semi-distributed, physically based continuous time-step environmental analysis software package designed to facilitate watershed hydrology and water quality simulation (Duda et al. 2012). It is one of the comprehensive watershed hydrology and water quality models that allows the integrated simulation of land and soil contaminant runoff processes with in-stream hydraulic and sediment-chemical interactions. The HSPF can be used to determine the fate and transport of contaminants in streams and lakes and to estimate nonpoint-source loads from different land uses. HSPF consists of three major modules: PERLND (Pervious Land Segment), IMPLND (Impervious Land Segment), and RCHRES (reach/reservoirs).

#### HSPF model segmentation and watershed delineation

**Fig. 2:**
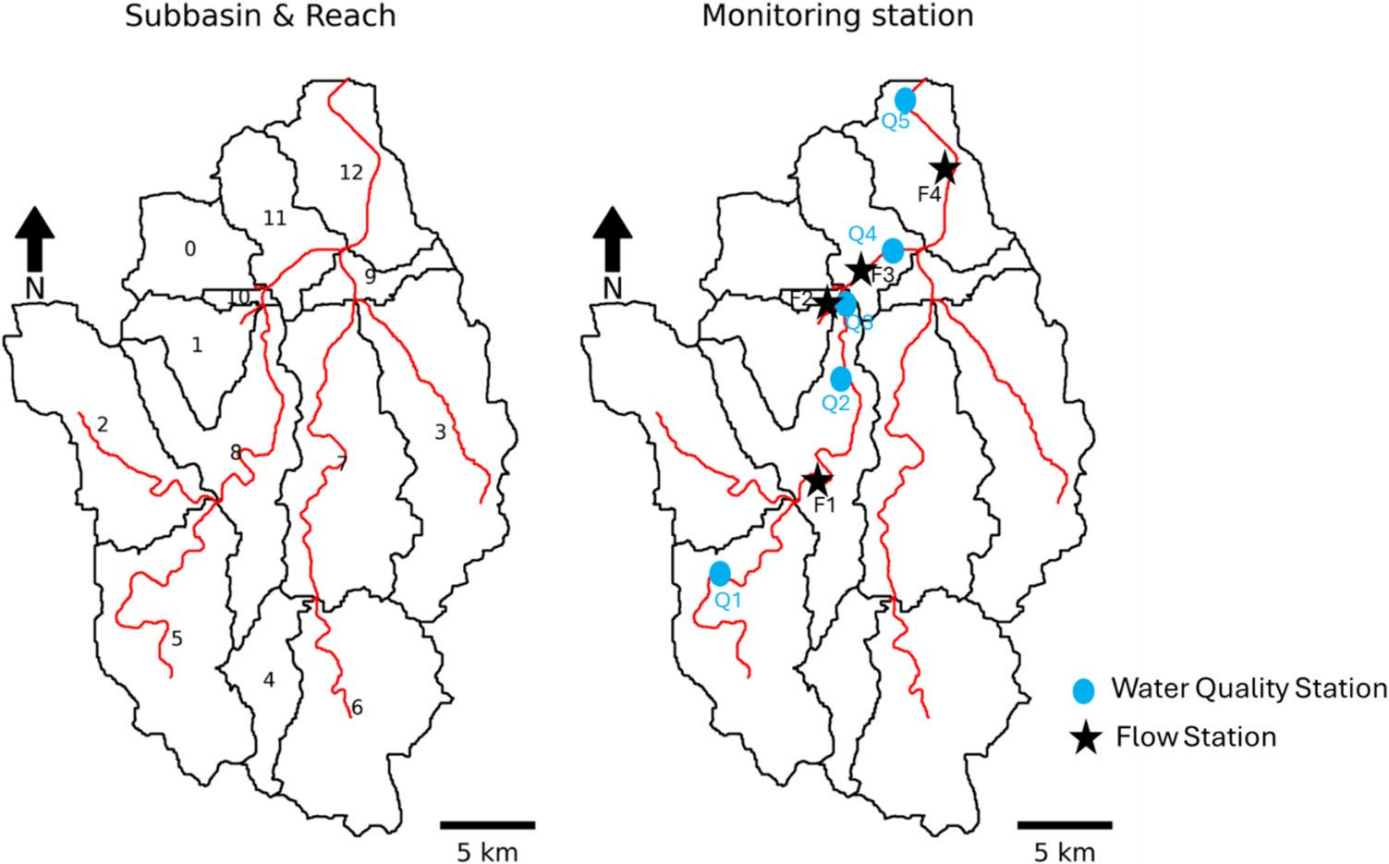
Delineated subbasins and reaches with the water quality and flow station

The accuracy of model output is dependent on the spatial and temporal sizes of discretization. A Thiessen polygon network was used to accurately simulate the model as shown in Fig. 1d. According to the Thiessen polygon, there are six gauging stations in the Gap-Cheon watershed. Therefore, the watershed was divided into six meteorological segments, or met segments, according to the covering area of the rain gauging station. As a result, six different hourly precipitation data were used in the watershed. The HSPF model was run using automatic watershed delineation. As a result, thirteen subbasins and reaches were delineated, as shown in Fig. 2.

#### HSPF model calibration

Model calibration is an iterative process involving adjusting parameters in the range of their variation. To consider a model as a well-calibrated model, it must have a satisfactory level of agreement between observed and simulated parameter values. This degree of agreement could be measured using statistical metrics. In this study, we used *R*^2^, percent bias (PBIAS), and mean absolute error (MAE).

### FLUS model

For land use prediction, this study utilized the FLUS model to predict changes in land use types (Liu et al. 2017). This is achieved using the top-down System Dynamics (SD) and bottom-up Cellular Automata (CA) methods. The FLUS model employs multiple feature variables to simulate future land use patterns. For this study, we prepared seven variables, namely, aspect, elevation, slope, Normalized Difference Vegetation Index (NDVI), Normalized Difference Built-up Index (NDBI), Normalized Difference Water Index (NDWI), and distance to roads network. For elevation-related variables (aspect and slope), data are obtained from the digital elevation model (DEM) provided by the National Aeronautics and Space Administration (NASA), which was obtained from the shuttle radar topography mission (SRTM). For spectral indices (NDVI, NDBI, and NDWI), we used Landsat 8 mean images for the year 2022, and for the road network, we obtained the shapefile from the humanitarian data exchange site (data.humdata.org).

The FLUS model is based on the spatial simulation method that utilizes a CA model and trains an Artificial Neural Network (ANN) model to create a probability-of-occurrence (PO) surface for different land use types (Liu et al. 2021). The links between historical land use and various driving factors are defined using an ANN. Changes in land-use distribution are guided by the PO surfaces obtained from the ANN. The CA model’s total likelihood of land-use type is adjusted according to the total quantity of that land-use area in the scenario using a self-adaptive inertia coefficient. Overall, the simulation process is divided into several periods, and the bottom-up CA model and the top-down urban demand forecasting model are strongly linked during the modeling. A multiple CA allocation model is established to simulate the future spatial pattern based on the land-use demands determined by the SD model. The CA simulation is carried out in two steps: (1) an ANN is used to train and evaluate the likelihood of each land-use type occurring on a specific grid cell, and (2) sophisticated self-adaptive inertia and competition mechanisms are developed to address competition and interactions among the different land-use types.

Lastly, the SD model is used to compute future land use scenario demands based on various factors, including socio-economic and natural environmental factors at national/regional scales. Self-adaptive inertia and competition mechanisms are developed within the CA model to handle the complex competitions and interactions among the different land-use types. The aggregate probability of all land-use types at each grid cell is estimated in these two steps, and the dominant land-use type is assigned to the cell during the CA iteration. Depending on their combined probability and the roulette pick, a particular land grid remains the present land-use type or evolves into another type during the allocation process.

## Result and discussion

### Land use change

In this study, land use data for the years 2012 and 2022 were used and analyzed to see how the changes affect the hydrology and the water quality in the Gap-Cheon watershed. Moreover, based on the land use of 2012 and 2022, we predicted the 2052 land use using the FLUS model and analyzed its effect on the watershed hydrology and water quality. In terms of the accuracy of the FLUS model, conventionally, the FLUS simulation accuracy depends on the model’s hidden layers and several iterations of the model through which the models learn the patterns in the input data. To predict the land use for 2052, the FLUS model was specifically run with 12 hidden layers of a neural network and with 300 iterations. With these settings, the FLUS model achieved a root mean square error (RMSE) accuracy of 0.26479, ensuring that the model is well-trained and accurate for simulating future land use dynamics based on the provided land use layers and features.

In the Gap-Cheon watershed, there are seven land use classes, namely, urban, agriculture, forest, grass, wetland, barren, and water, as shown in Fig. S1. Table S1 shows the area of each land use class in the watershed for 2012, 2022, and 2052. In 2012, the largest urban area was identified in subbasin 3, with 25 km^2^, followed by subbasin 7 and 12, with 19 km^2^ and 14 km^2^, respectively, as shown in Fig. S2. Subbasin 6 had the largest agricultural area in 2012, and this has changed to subbasin 5 in 2022 and 2052 with 17.7 km^2^, 10.46 km^2^, and 9.2 km^2^, respectively, as shown in Fig. S3. On the other hand, subbasin 5 has the largest forest area in all the years (Fig. S4). The largest wetland area has also shown a consistent trend throughout the years, where the maximum area is in subbasin 8, as shown in Fig. S5. A great change in land use was recorded in all years. Urban, agricultural, and forest land have significantly reduced by 8%, 27%, and 1%, respectively, in 2022 compared to 2012. On the other hand, grass and wetlands have increased by 132% and 37%, respectively. Under the urban land class, there are six subclasses: residential, industrial, commercial, recreational, road, and public facilities. The residential area is the one that shows profound change. It has reduced by 26% in 2022. The highest reduction in the residential area is in subbasins 4, 5, and 6. The decline in the residential area might be directly related to the decrease in the number of populations in the watershed. The population has declined greatly from 2009 to 2021, as shown in Fig. S6. This might be because of migration to the city and the decline in the birth rate (Yun et al. 2022). Industrial and public facilities have also reduced by 15.7% and 23.8%, respectively. On the other hand, commercial and roads increased by 40% and 18%, respectively. In most subbasins, commercial facilities were increased in 2022, majorly in subbasins 12, 1, and 8. Additionally, more roadways were increased in subbasins 5, 6, and 8. Under agricultural area, rice paddy area has reduced from 39.9 to 14.0 km^2^ in 2022. In the same way, the field area was also reduced from 53 to 46 km^2^. Conversely, cultivated orchard land and other agricultural areas have shown an increasing trend. Broad-leaved, coniferous, and mixed forests are the subclass of forest land. In this category, the broad-leaved forest increased by almost 50% in 2022, but coniferous and mixed forests showed a significant decline in 2022 compared to 2012 by 28.2% and 26.2%, respectively. The wetland area has also increased in the watershed by 37.3%, where the maximum increase was shown in subbasins 12, 6, and 10.

Based on the land use of 2012 and 2022, we predicted the change in the land use for 2052 using the FLUS model. The result shows that the urban area is expected to decrease in 2052 by 8.9%, majorly in subbasins 4, 5, and 6. Agricultural land use is also predicted to decrease by 17.9%, with a major decline of 27%, 26.5%, and 26.5% in subbasins 6, 4, and 9, respectively. On the other hand, the forestland, grassland, and wetland areas are predicted to increase by 2.2%, 18.6%, and 4.3%, respectively, in 2052. Details of predicted land use change for 2052 compared to 2022 are given in Fig. S7.

### Model calibration and performance of stream flow and water temperature

**Fig. 3:**
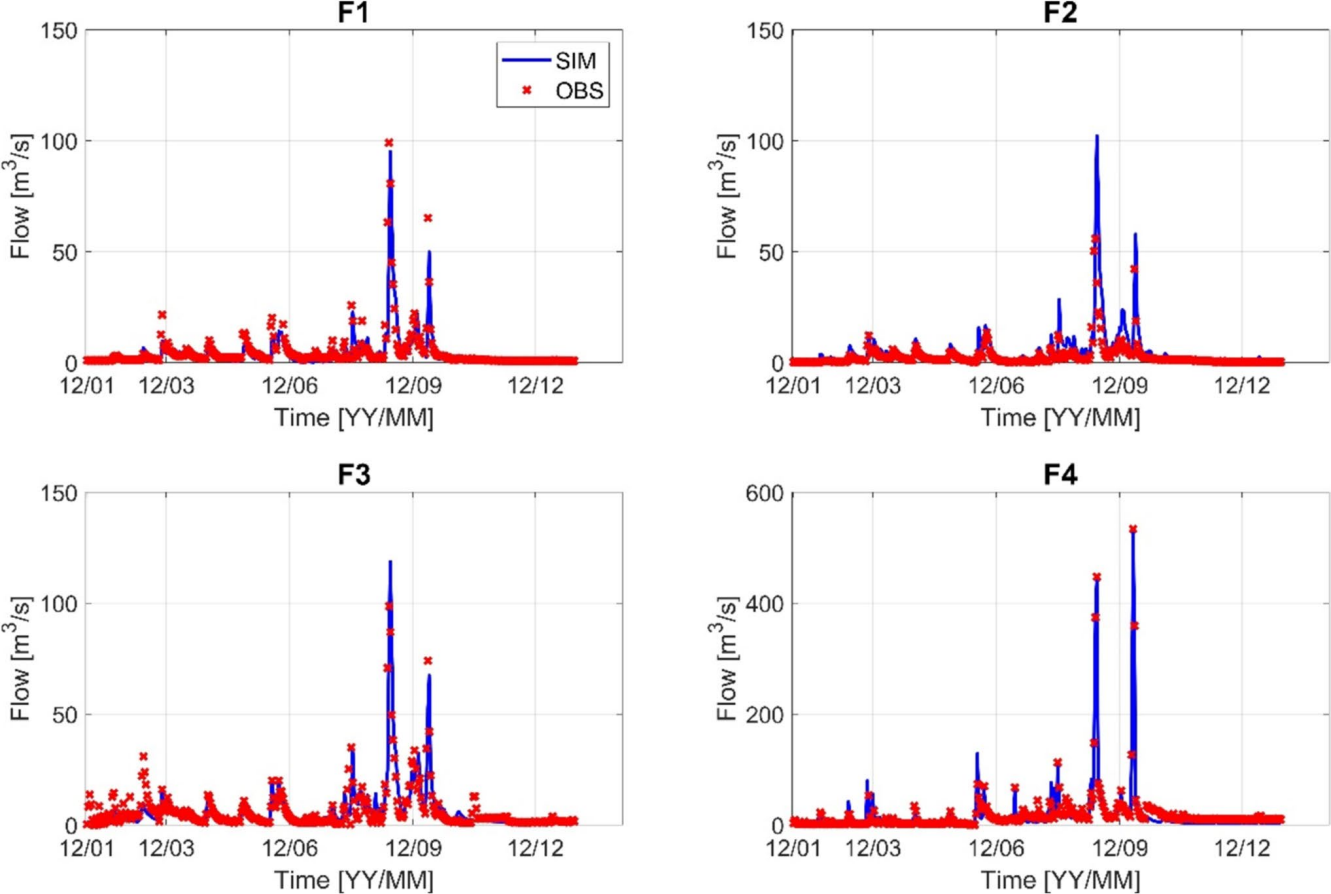
Stream flow calibration result (F1–F4) for the year 2012

**Fig. 4:**
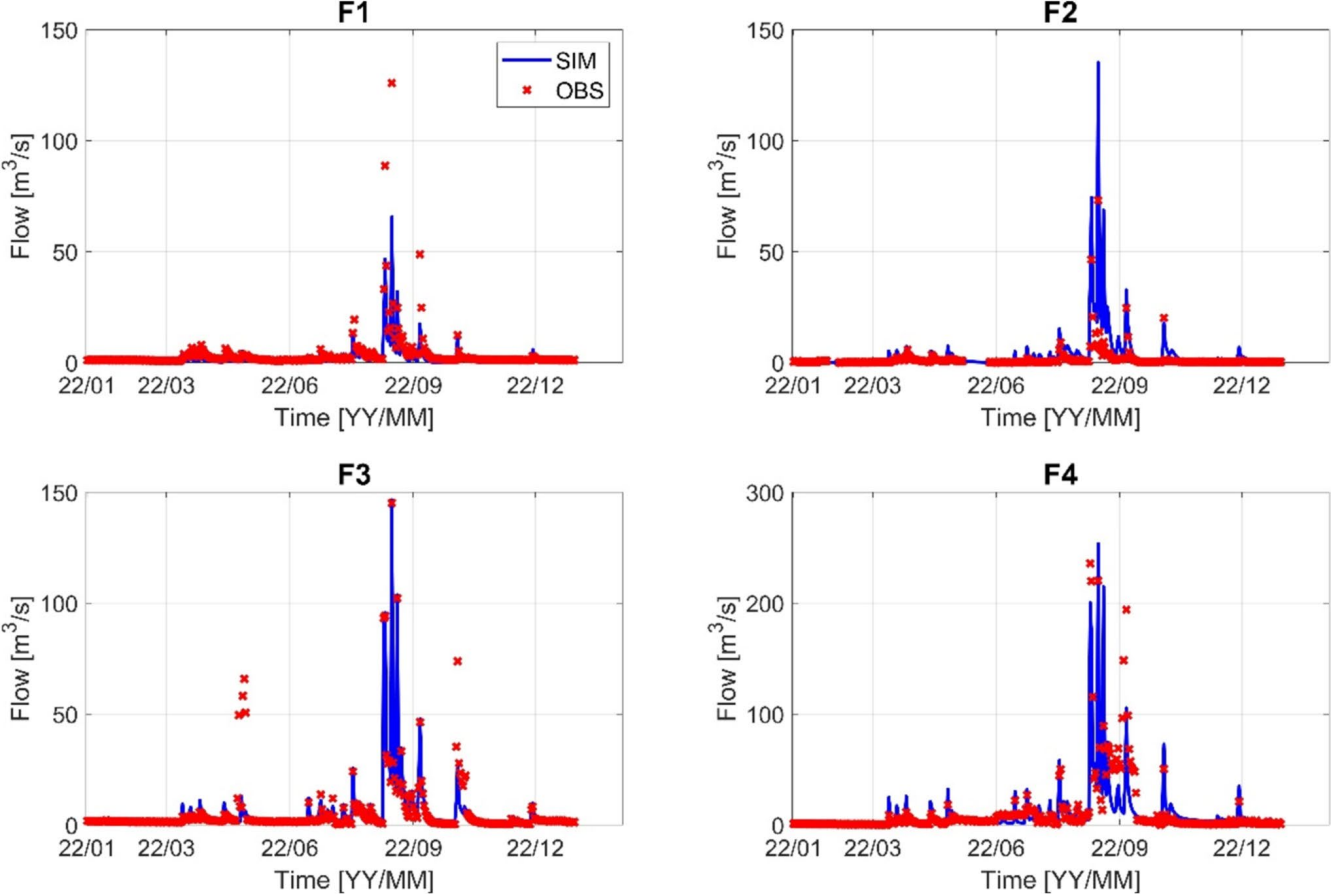
Stream flow calibration result (F1–F4) for the year 2022

**Fig. 5:**
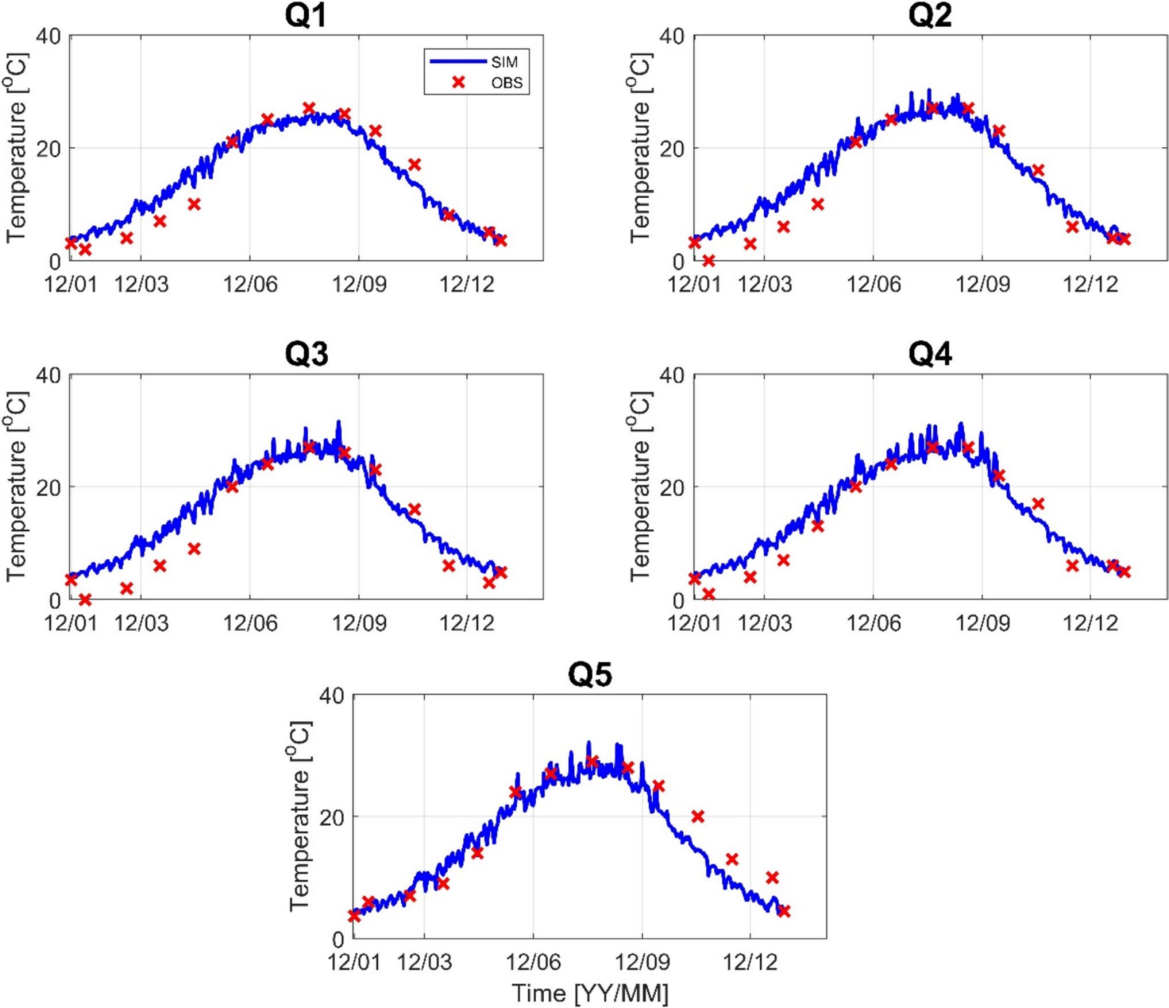
Water temperature calibration result (Q1–Q5) for the year 2012

**Fig. 6:**
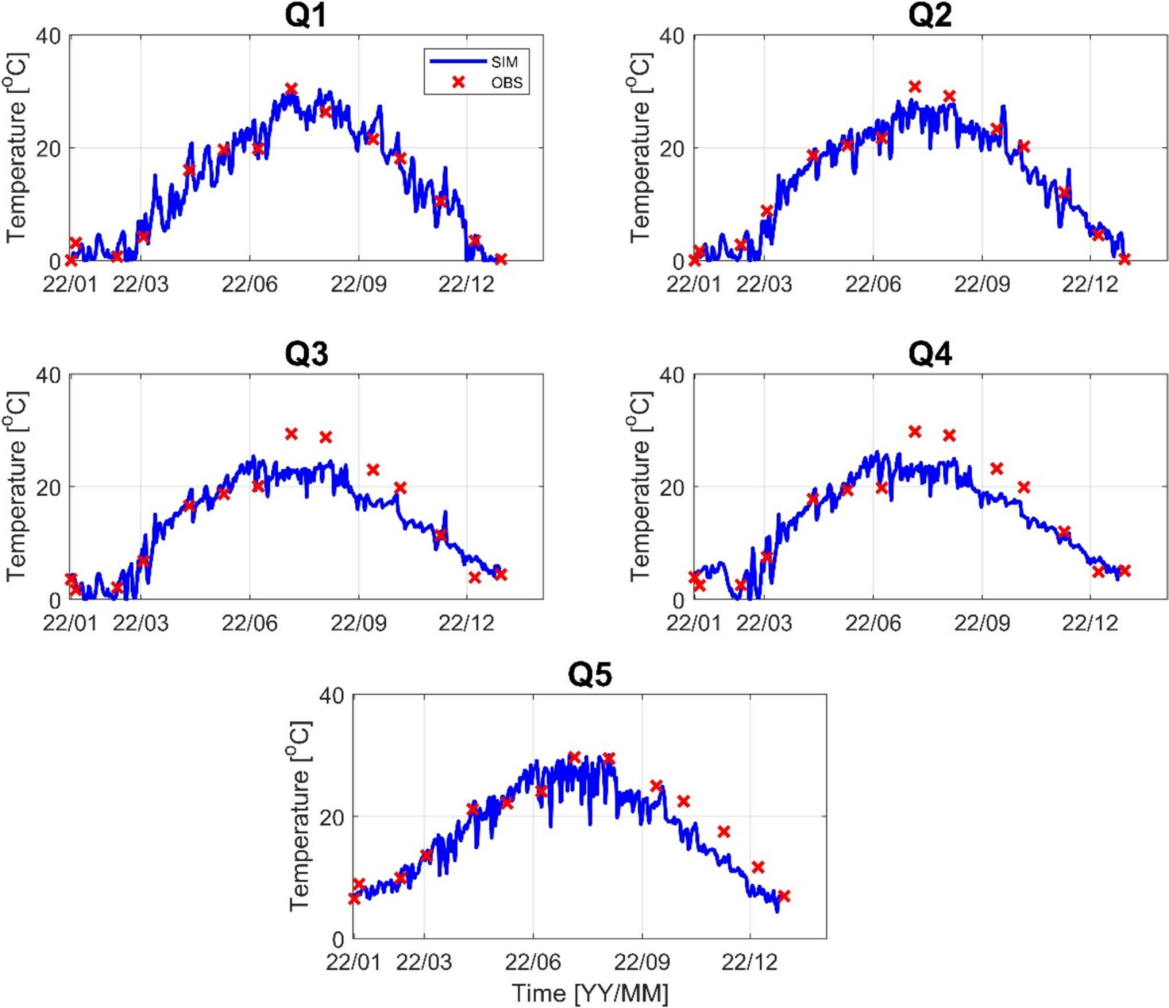
Water temperature calibration result (Q1–Q5) for the year 2022

Before running the HSPF, model calibration and verification were made. Stream flow and water temperature were calibrated first, followed by calibration of water quality variables: biological oxygen demand (BOD), total nitrogen (TN), and total phosphorus (TP). Stream flow was calibrated at four flow monitoring stations, and water temperature was calibrated at five monitoring stations. Figures 3 and 4 show the performance of stream flow at four flow monitoring stations for 2012 and 2022, respectively. The simulated results are in good agreement with the observed data in all monitoring stations. Most of the calibrated results have an *R*^2^ value greater than 0.9 and a PBIAS of less than 18% for 2012, an *R*^2^ value greater than 0.7, and a PBIAS value of less than 20% for 2022. In the same way, the water temperature was calibrated at five monitoring stations, as shown in Figs. 5 and 6. For 2012, most of the *R*^2^ has a value greater than 0.94 and PBIAS less than 10%. For 2022, the *R*^2^ is greater than 0.9, and PBIAS is less than 9%. The statistical metrics for flow and temperature calibration results are given in Table 1 for 2012 and 2022, respectively.

**Table 1:** Statistical performance results for stream flow and water temperature of 2012 and 2022

Variable	Site	2012			2022		
		*R*^2^	PBAIS	MAE	*R*^2^	PBAIS	MAE
Stream flow	F1	0.94	17.43	2.33	0.90	27.96	3.40
	F2	0.90	16.5	2.3	0.84	14.8	2.55
	F3	0.82	6.33	2.5	0.80	11.77	1.96
	F4	0.95	12.0	2.4	0.70	8.95	8.95
Water temperature	Q1	0.96	1.4	0.06	0.96	0.09	0.44
	Q2	0.96	8.41	0.07	0.96	7.44	0.43
	Q3	0.95	12.74	0.09	0.89	8.02	0.42
	Q4	0.95	6.56	0.06	0.89	7.32	0.44
	Q5	0.92	9.25	0.07	0.92	9.4	0.54

### Model calibration and performance of water quality

For the water quality variables, model calibration and performance were done at five water quality monitoring stations (Q1–Q5) for both the years 2012 and 2022. The water quality variables used in this study were BOD, TN, and TP. Model parameters were adjusted iteratively within a feasible range to achieve the best agreement between simulated and observed values. Figure S8 shows the graphical comparison of observed and simulated results for 2012. The result shows a reasonable model fit in all water quality monitoring stations. In the case of TP, it was over-predicted at the Q1 station in the wet season. Figure S9 illustrates the water quality calibration result for the year 2022. The result shows a fair model fit from Q1 to Q5 stations. In the same way, as in 2012, TP was also over-predicted at the Q2 station during the high flow season.

### Influences of land use change on water quantity

**Fig. 7:**
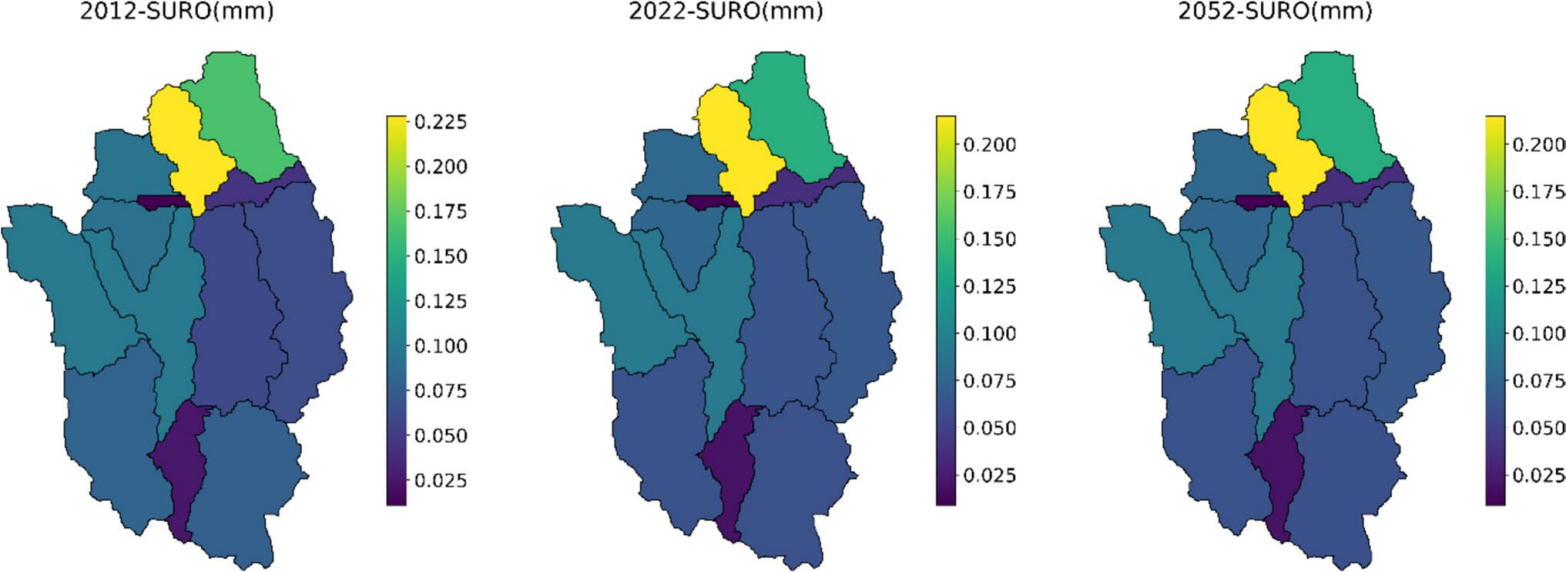
Changes in surface runoff (SURO) at each subbasin for 2012, 2022, and 2052

Based on the changes in land use in 2012, 2022, and 2052, we calculated the changes in water quantity across the watershed. The spatial variations of surface runoff (SURO) and total evapotranspiration (TAET) were modeled for each subbasin. SURO is characterized by a maximum decrease of up to 21% at subbasin 4 and 6 and 16% at subbasin 9 in 2022. The urban land has greatly decreased in these subbasins, especially subbasins 4 and 6. In contrast, the grassland (urban green space) has increased in these subbasins. In subbasins 4, 6, and 9, the urban land was reduced by 51%, 13%, and 9%, respectively. In contrast, the grassland increased by 380%, 862%, and 167%, respectively. Urban areas contribute to increased surface runoff due to the extensive development and transformation of natural landscapes. Urban areas are characterized by the extensive use of impervious surfaces that do not allow water to penetrate the ground, leading to increased runoff (Sun et al. 2014). The reduction of urban areas can decrease surface runoff through several mechanisms, primarily by allowing more natural processes to regulate water flow. Reintroducing vegetation and green space in reduced urban areas can enhance water absorption. Plants and trees absorb rainwater, and their root systems promote soil permeability, reducing surface runoff. The grassland can absorb and hold more water, reducing the amount of water available for runoff. This indicates that green space has effectively controlled most of the surface runoff by infiltrating the runoff. The green space as green infrastructure functions through the process of infiltration, storage, and interception in the water cycle system (Jia et al. 2016). The effectiveness of urban green spaces in reducing flooding has been demonstrated in runoff management strategies (Kim et al. 2016). According to the predicted land use change of 2052, the SURO is also expected to decrease in subbasins 4 and 6 by 11%. In most subbasins, surface runoff greatly decreased in both 2022 and 2052, as shown in Fig. 7.

**Fig. 8:**
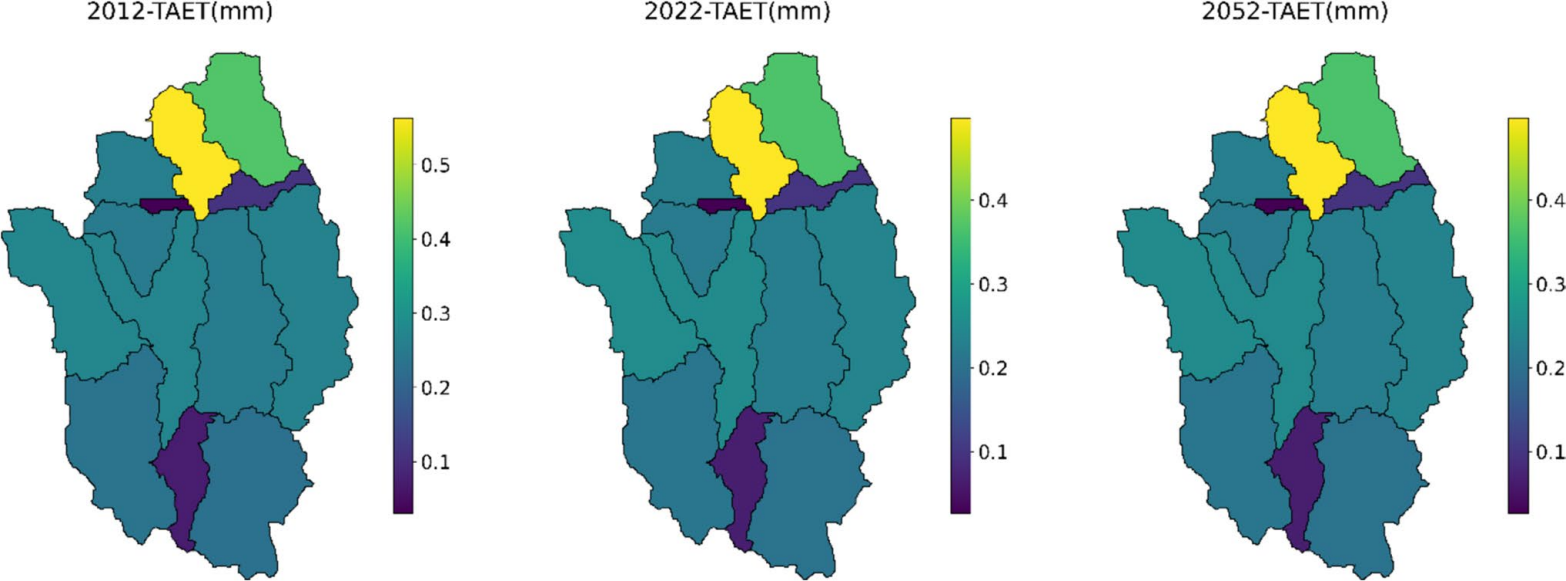
Changes in total evapotranspiration (TAET) at each subbasin for 2012, 2022, and 2052

The evapotranspiration (TAET) has decreased in all subbasins, with a higher reduction of 12.4% and 63.5% at subbasin 9 in 2022 and 2052, respectively (Fig. 8). As evapotranspiration is the process by which water is transferred from the soil and plant to the atmosphere through evaporation and transpiration (Ghiat et al. 2021), the decrease in TAET might have some possible reasons. Trees and forests play a crucial role in transporting water through their leaves, and deforestation in the Gap-Cheon watershed might reduce this process. Forest area in subbasin 9 was reduced by 9% due to deforestation. This significantly affected evapotranspiration. In addition, the decrease in agricultural land and changes in farming practices can also influence evapotranspiration. Land management, such as irrigation and drainage can also alter the hydrological balance, affecting evapotranspiration in the watershed. Alterations in agricultural practices, such as increased irrigation, changes in crop types, or land conversion from natural vegetation to croplands, could affect evapotranspiration rates. Certain agricultural practices might reduce water availability for evapotranspiration. Moreover, in this subbasin, the urban area increased by 2%, which can directly impact evapotranspiration. Urban areas typically have a high percentage of impervious surfaces such as asphalt, concrete, and buildings. These surfaces prevent water from infiltrating into the soil, reducing the amount of water available for plant uptake and subsequent transpiration.

**Fig. 9:**
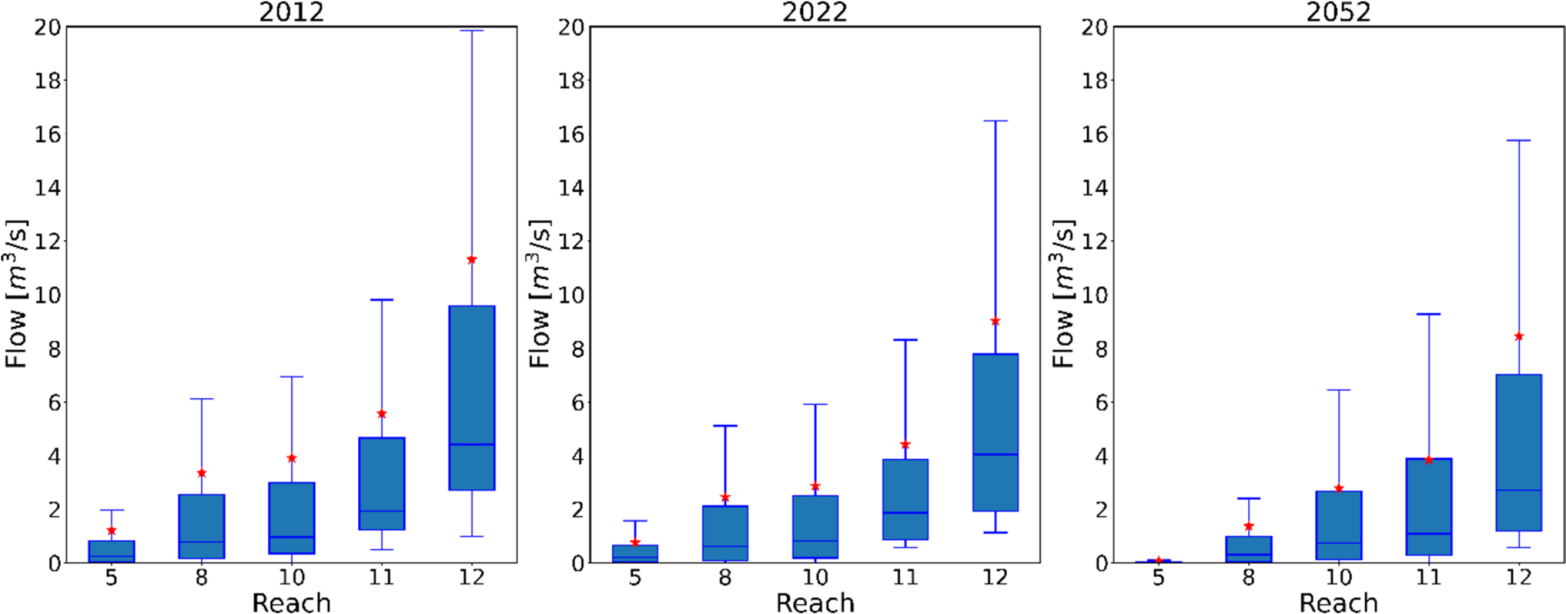
Stream flow variation across the catchment

The changes in land use not only affected the SURO and TAET but also exerted a profound effect on the stream flow. In 2012, the mean flow at reach 5 was 1.21 m^3^/s but decreased to 0.76 m^3^/s in 2022 and further to 0.06 m^3^/s in 2052 as illustrated in Fig. 9. At reach 8, the stream flow was 3.36 m^3^/s in 2012, experiencing a substantial reduction of 27% and 59% in 2022 and 2052, respectively. Similar trends were observed in reach 10 and 12. At reach 10, it has reduced by 26% and 28% in 2022 and 2052. Notably, reach 12, which had the highest flow rate of 11.30 m^3^/s also reduced to 9.03 m^3^/s and 8.46 m^3^/s in 2022 and 2052, respectively. This indicates that the reduction in urban areas and the increase in green spaces have reduced the flows to the streams from the watershed. The increase in wetlands also has contributed to reducing stream flows by holding water before entering the streams.

### Influences of land use change on water quality

Changes in BOD, TN, and TP were analyzed for all the changes in the land use of 2012, 2022, and 2052. BOD is the most crucial water quality variable for assessing water pollution. It indicates the presence of organic matter in the water. Most organic matters originate from urban areas, especially from combined sewer overflows (Legesse et al. 2022). The pollutant load of BOD has greatly declined in both 2022 and 2052 compared to 2012 in all subbasins (Fig. S10). This indicates that the change in urban areas has reduced the transport of organic matter. The maximum BOD load was identified in subbasin 3 with 1314.2 kg/d, 628 kg/d, and 616.18.3 kg/d in 2012, 2022, and 2052, respectively. The BOD load was reduced in this subbasin by 52.2% and 1.89% in 2022 and 2052, respectively. Subbasin 3 has the largest urban area among the others. However, the urban area has reduced by 7.6% and 4.1% in 2022 and 2052, respectively. On the other hand, the grassland (urban green space) has increased by 222% and 18% in 2022 and 2052. This indicates that the conversion of some urban areas with green space has greatly influenced the BOD load. It clearly shows that green space acts as a natural filter that can be considered a nature-based solution. When runoff water flows through this green space, it absorbs and traps nutrients and organic matter, preventing them from reaching water bodies. In addition, the soil in green spaces has greater porosity and permeability compared to paved surfaces in urban areas. The microorganisms in green space soil play a significant role by breaking down organic matter and converting it into less harmful substances.

**Fig. 10:**
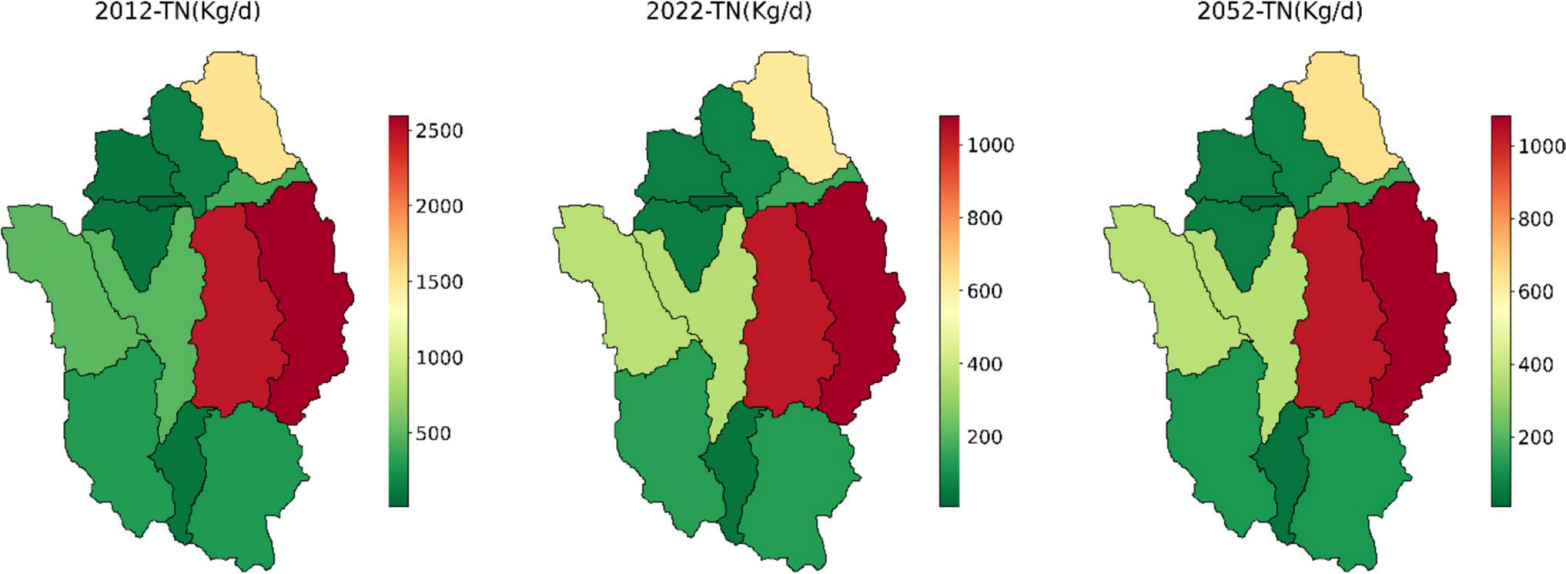
Changes in TN load at each subbasin for 2012, 2022, and 2052

**Fig. 11:**
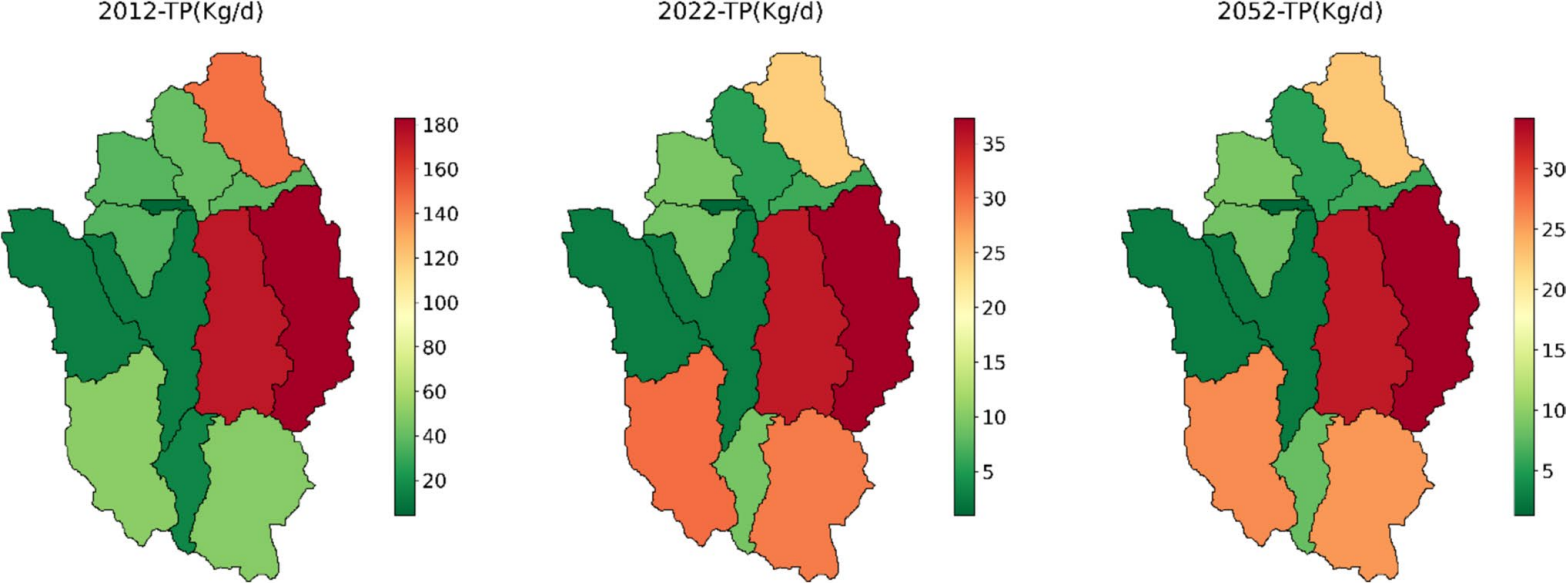
Changes in TP load at each subbasin for 2012, 2022, and 2052

The model result also revealed that land use changes also affected the catchment’s nutrient load. Almost in all subbasins, TN and TP load has shown a pronounced decline in 2022 and 2052, as shown in Figs. 10 and 11. The highest TN and TP load reduction was recorded in subbasins 9, 12, and 3. In these subbasins, the TN load was reduced by almost 60%, and the TP load was reduced by more than 80% in 2022. In these three subbasins, agricultural land has been significantly reduced, which leads to the reduction of nutrients from this land. In addition, total nitrogen (TN) and total phosphorus (TP) in subbasins 9, 12, and 13 were reduced by 5% and 8%, respectively, for the predicted year 2052. This is attributed to a substantial reduction in agricultural activities and an increase in forested areas within these specific subbasins. Agriculture is one of the main sources of water pollution in many regions, more than urban and industrial pollution sources (Mateo-Sagasta et al. 2017). Agricultural water pollution poses a direct threat to human health, manifesting in conditions like blue-baby syndrome, where elevated nitrate levels in water can lead to methemoglobinemia in infants (Mateo-Sagasta et al. 2017). The high use of fertilizers and pesticides can also pose a great threat to water quality and human health. Therefore, the reduction of agricultural land has a great effect on reducing nutrient loads and improving the health of humans. The increase in forested areas or the revegetation of the subbasins also has a great effect. The revegetation of riparian and riverbanks profoundly influences fluvial geomorphology by impacting resistance to flow and sediment (Webb and Erskine 2003). The forest area (revegetated area) also can trap nutrients that are exported from agricultural areas. The presence of vegetation could act as a natural protection against the impact of land use change. The presence significantly contributes to the resilience of the watershed by stabilizing soil, regulating water flow, and retaining nutrients. Moreover, constructed wetlands have increased exponentially in these subbasins. Wetlands are one of the nature-based solutions to mitigate floods and improve the water quality of waterways. The expansion of wetlands acts as a natural filter by providing additional retention time (Richardson et al. 2011). Figure S11 shows a great increase in wetlands in subbasin 12 and 3. Due to their long hydraulic residence time, they are commonly referred to as natural sponges, which play a significant role in reducing peak flows, nutrients, and erosion rates (Gibbs 2000; Seifollahi-Aghmiuni et al. 2019). Previous studies also revealed that constructed wetlands have reduced nutrients and organic matter greatly (Adeola et al. 2009; Lin et al. 2020).

## Conclusions

In conclusion, this paper explores the relationship between land use changes and their impacts on hydrological processes and water quality in the Gap-Cheon watershed. It includes historical land use analysis and future predictions using the FLUS model, as well as examines the effects on water quantity and quality. The study reveals shifts in urbanization, agriculture, and forested areas, predicting further changes by 2052. It highlights the decrease in urban areas and increases in wetlands and green spaces, impacting surface runoff, evapotranspiration, and nutrients. The findings underscore the dynamic nature of land use changes and their complex interplays.

Urban green spaces effectively mitigate the negative impacts of urbanization on water quantity and quality by reducing surface runoff, absorbing nutrients, and enhancing soil permeability. These nature-based solutions not only improve urban aesthetics but also play a crucial role in sustainable watershed management. Forests, with their root systems and transpiration processes, are vital in regulating the water balance. Deforestation reduces total evapotranspiration, affecting the hydrological cycle, while the increase in broad-leaved forests and forested areas supports water availability and acts as buffers against erosion and nutrient runoff. Overall, both urban green spaces and forests are essential for maintaining watershed health and resilience. Wetlands serve as natural sponges, vital in flood mitigation and enhancing water quality. Their expansion in certain subbasins reduces nutrient and organic matter loads by prolonging hydraulic residence time, which enhances pollutant retention. As natural filters, wetlands also help lower peak flows and reduce erosion rates. This study underscores the importance of conserving and restoring wetlands as effective nature-based solutions for sustainable watershed management.

In conclusion, this paper enhances our understanding of the complex interactions between land use changes, hydrological processes, and water quality. It emphasizes the critical role of urban green spaces, forests, and wetlands in mitigating human impacts on watersheds. Conservation and sustainable management of these natural features are essential for creating resilient ecosystems capable of withstanding the challenges of changing land use patterns. Integrating nature-based solutions into land use planning is vital for maintaining the long-term health and functionality of watersheds such as the Gap-Cheon watershed.

## Supplementary Information

Below is the link to the electronic supplementary material.

Supplementary file1 (DOCX 11.5 MB)

## Data Availability

The data used in this study are from the Korean National database system. Weather data is available at data.kma.go.kr. The digital elevation model is available at ngii.go.kr. Land use data is available at egis.me.go.kr. Water quantity and quality data are available at wamis.go.kr.

## References

[CR1] Adeola S, Revitt M, Shutes B, Garelick H, Jones H, Jones C (2009) Constructed wetland control of BOD levels in airport runoff. Int J Phytorem 11(1):1–10

[CR2] Albek M, Öğütveren ÜB, Albek E (2004) Hydrological modeling of Seydi Suyu watershed (Turkey) with HSPF. J Hydrol 285(1–4):260–271

[CR3] Aquilué N, De Cáceres M, Fortin M-J, Fall A, Brotons L (2017) A spatial allocation procedure to model land-use/land-cover changes: accounting for occurrence and spread processes. Ecol Model 344:73–86

[CR4] Chen Y, Xu C-Y, Chen X, Xu Y, Yin Y, Gao L, Liu M (2019) Uncertainty in simulation of land-use change impacts on catchment runoff with multi-timescales based on the comparison of the HSPF and SWAT models. J Hydrol 573:486–500

[CR5] Chen Z, Huang M, Zhu D, Altan O (2021) Integrating remote sensing and a Markov-FLUS model to simulate future land use changes in Hokkaido. Japan Remote Sens 13(13):2621

[CR6] Crawford NH, Linsley RK (1966) Digital simulation in hydrology. Stanford Watershed Model IV. Technical Report No. 39, Department of Civil and Environmental Engineering, Stanford University, Stanford

[CR7] Duda PB, Hummel PR, Donigian AS Jr, Imhoff JC (2012) BASINS/HSPF: model use, calibration, and validation. Trans ASABE 55(4):1523–1547

[CR8] Ghiat I, Mackey HR, Al-Ansari T (2021) A review of evapotranspiration measurement models, techniques and methods for open and closed agricultural field applications. Water 13(18):2523

[CR9] Gibbs JP (2000) Wetland loss and biodiversity conservation. Conserv Biol 14(1):314–317

[CR10] Jia Z, Tang S, Luo W, Li S, Zhou M (2016) Small scale green infrastructure design to meet different urban hydrological criteria. J Environ Manage 171:92–100 26831449 10.1016/j.jenvman.2016.01.016

[CR11] Kim TG, Choi K-S (2020) A study on water quality change by land use change using HSPF. Environ Eng Res 25(1):123–128

[CR12] Kim H, Lee D-K, Sung S (2016) Effect of urban green spaces and flooded area type on flooding probability. Sustainability 8(2):134

[CR13] Legesse NS, Kim J, Seo D (2022) Evaluation of significant pollutant sources affecting water quality of the Geum River using principal component analysis. J Korea Water Resour Assoc 55(8):577–588

[CR14] Li Z, Liu H, Luo C, Li Y, Li H, Pan J, Jiang X, Zhou Q, Xiong Z (2015) Simulation of runoff and nutrient export from a typical small watershed in China using the Hydrological Simulation Program-Fortran. Environ Sci Pollut Res 22:7954–7966 10.1007/s11356-014-3960-y 25516253

[CR15] Lin CJ, Chyan JM, Zhuang WX, Vega FA, Mendoza RMO, Senoro DB, Shiu RF, Liao CH (2020) Application of an innovative front aeration and internal recirculation strategy to improve the removal of pollutants in subsurface flow constructed wetlands. J Environ Manage 256:109873 31822455 10.1016/j.jenvman.2019.109873

[CR16] Liu X, Liang X, Li X, Xu X, Ou J, Chen Y, Li S, Wang S, Pei F (2017) A future land use simulation model (FLUS) for simulating multiple land use scenarios by coupling human and natural effects. Landsc Urban Plan 168:94–116

[CR17] Liu P, Hu Y, Jia W (2021) Land use optimization research based on FLUS model and ecosystem services–setting Jinan City as an example. Urban Climate 40:100984

[CR18] Mateo-Sagasta J, Marjani Zadeh S, Turral H, Burke J (2017) Water pollution from agriculture: A global review. Executive summary. Food and Agriculture Organization of the United Nations (FAO) and International Water Management Institute (IWMI)

[CR19] Namugize JN, Jewitt G, Graham M (2018) Effects of land use and land cover changes on water quality in the uMngeni river catchment, South Africa. Physics Chem Earth Parts a/b/c 105:247–264

[CR20] Owuor SO, Butterbach-Bahl K, Guzha AC, Rufino MC, Pelster DE, Díaz-Pinés E, Breuer L (2016) Groundwater recharge rates and surface runoff response to land use and land cover changes in semi-arid environments. Ecol Process 5(1):1–21

[CR21] Ren Y, Lü Y, Comber A, Fu B, Harris P, Wu L (2019) Spatially explicit simulation of land use/land cover changes: current coverage and future prospects. Earth Sci Rev 190:398–415

[CR22] Richardson CJ, Flanagan NE, Ho M, Pahl JW (2011) Integrated stream and wetland restoration: a watershed approach to improved water quality on the landscape. Ecol Eng 37(1):25–39

[CR23] Roostaee M, Deng Z (2019) HSPF-based watershed-scale water quality modeling and uncertainty analysis. Environ Sci Pollut Res 26:8971–8991 10.1007/s11356-019-04390-0 30719665

[CR24] Sapena M, Ruiz L (2015) Analysis of urban development by means of multi-temporal fragmentation metrics from LULC data. Int Arch Photogramm Remote Sens Spat Inf Sci 40:1411–1418

[CR25] Seifollahi-Aghmiuni S, Nockrach M, Kalantari Z (2019) The potential of wetlands in achieving the sustainable development goals of the 2030 Agenda. Water 11(3):609

[CR26] Shiferaw N, Kim J, Seo D (2023) Identification of pollutant sources and evaluation of water quality improvement alternatives of a large river. Environ Sci Pollut Res 30(11):31546–31560 10.1007/s11356-022-24431-5 36447103

[CR27] Sun Z, Li X, Fu W, Li Y, Tang D (2014) Long-term effects of land use/land cover change on surface runoff in urban areas of Beijing, China. J Appl Remote Sens 8(1):084596–084596

[CR28] Verburg PH, Overmars KP (2009) Combining top-down and bottom-up dynamics in land use modeling: exploring the future of abandoned farmlands in Europe with the Dyna-CLUE model. Landscape Ecol 24:1167–1181

[CR29] Verburg PH, Crossman N, Ellis EC, Heinimann A, Hostert P, Mertz O, Nagendra H, Sikor T, Erb K-H, Golubiewski N (2015) Land system science and sustainable development of the earth system: a global land project perspective. Anthropocene 12:29–41

[CR30] Wan R, Cai S, Li H, Yang G, Li Z, Nie X (2014) Inferring land use and land cover impact on stream water quality using a Bayesian hierarchical modeling approach in the Xitiaoxi River Watershed, China. J Environ Manage 133:1–11 24342905 10.1016/j.jenvman.2013.11.035

[CR31] Webb AA, Erskine WD (2003) A practical scientific approach to riparian vegetation rehabilitation in Australia. J Environ Manage 68(4):329–341 12877867 10.1016/s0301-4797(03)00071-9

[CR32] Wilson C, Weng Q (2010) Assessing surface water quality and its relation with urban land cover changes in the Lake Calumet Area, Greater Chicago. Environ Manage 45:1096–1111 20364379 10.1007/s00267-010-9482-6

[CR33] Yazdi MN, Ketabchy M, Sample DJ, Scott D, Liao H (2019) An evaluation of HSPF and SWMM for simulating streamflow regimes in an urban watershed. Environ Model Softw 118:211–225

[CR34] Yun J, Kim CY, Son SH, Bae CW, Choi YS, Chung SH (2022) Birth rate transition in the Republic of Korea: Trends and prospects. J Korean Med Sci 37(42):e311. 10.3346/jkms.2022.37.e311 10.3346/jkms.2022.37.e304 PMC9623034 36325608

