# Supplementary material for: Land use dynamics and their impact on hydrology and water quality of a river catchment: a comprehensive analysis and future scenario
Source: Environ Sci Pollut Res Int. 2025 Jan 25;32(7):4124–36. doi: 10.1007/s11356-025-35946-y (PMC11835986; doi:10.1007/s11356-025-35946-y)
Supplement: Supplementary file 1 — Supplementary file1 (DOCX 11.5 MB) [file 11356_2025_35946_MOESM1_ESM.docx]

**Land Use Dynamics and Their Impact on Hydrology and Water Quality of a River Catchment: A Comprehensive Analysis and Future Scenario**

Natnael Shiferaw^1*^, Lulit Habte^2^, Mirza Waleed^3^

^1^ Australian Rivers Institute, School of Environment & Science, Griffith University, Nathan, QLD 4111

^2^ Julius Kruttschnitt Mineral Research Center, Sustainable Minerals Institute, University of Queensland, Indooroopilly, QLD 4068, Australia

^3^ Department of Geography, Hong Kong Baptist University, Hong Kong SAR, China

*Corresponding author: [natnael.legesse@griffithuni.edu.au](mailto:natnael.legesse@griffithuni.edu.au)


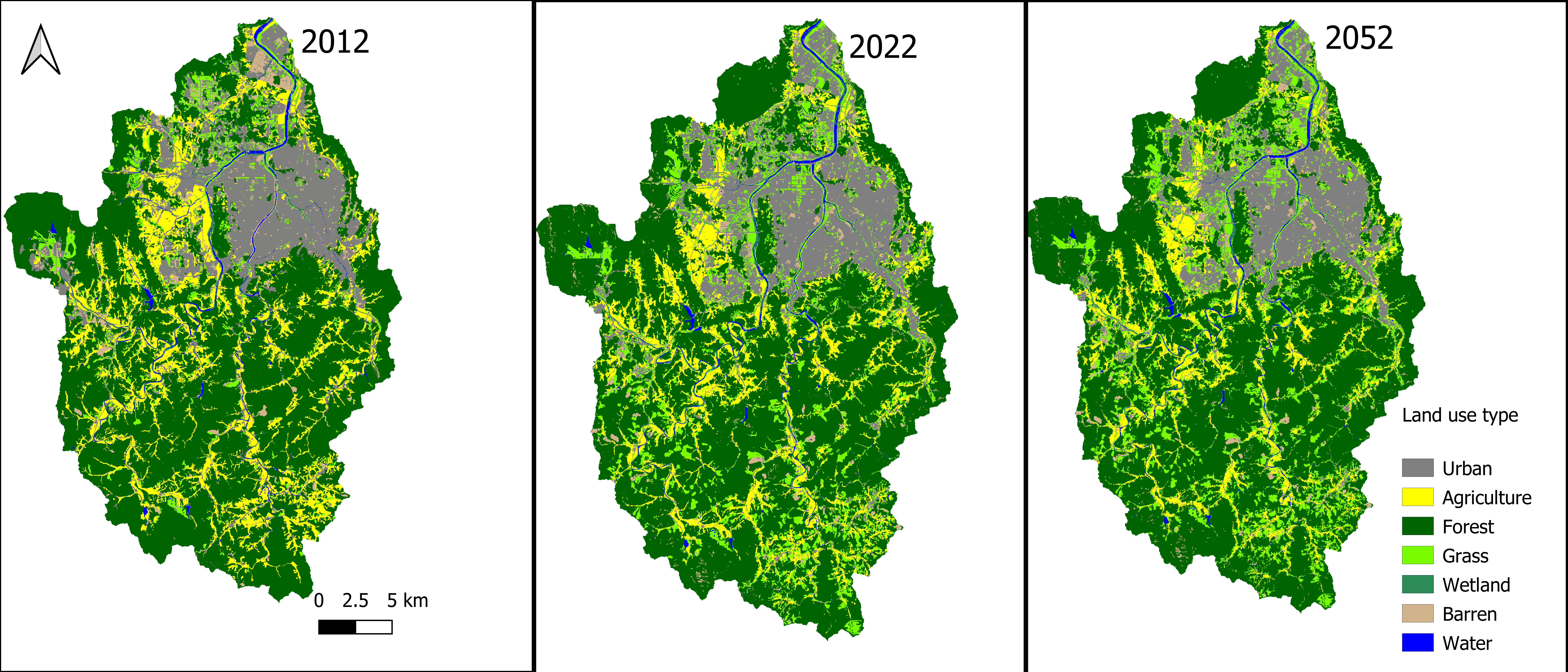


Fig.S1 Land use map of Gap-cheon watershed for the year 2012, 2022, and 2052

Table S1. Land use area of 2012, 2022 and 2052 for each class

| **Land use class** | **Area (Km^2^)**  **2012** | **Area (Km^2^)**  **2022** | **Area (Km^2^)**  **2052** |
| --- | --- | --- | --- |
| Urban | 108.78 | 100.07 | 91.07 |
| Agriculture | 101.16 | 73.68 | 60.92 |
| Forest | 363.14 | 359.61 | 367.71 |
| Grass | 32.74 | 76.02 | 90.19 |
| Wetland | 5.37 | 7.29 | 7.60 |
| Barren | 17.40 | 12.33 | 12.14 |
| Water | 8.81 | 7.24 | 6.88 |


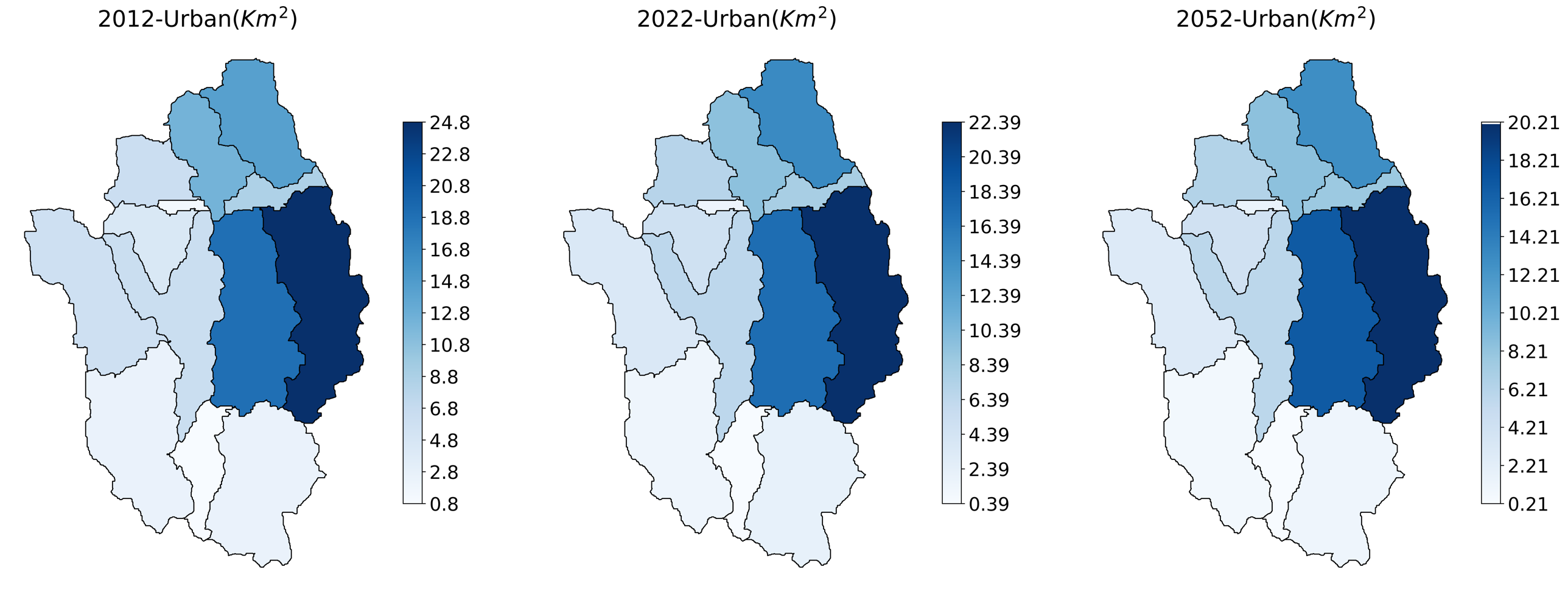


Fig. S2 Urban area at each subbasin for 2012, 2022, and 2052.


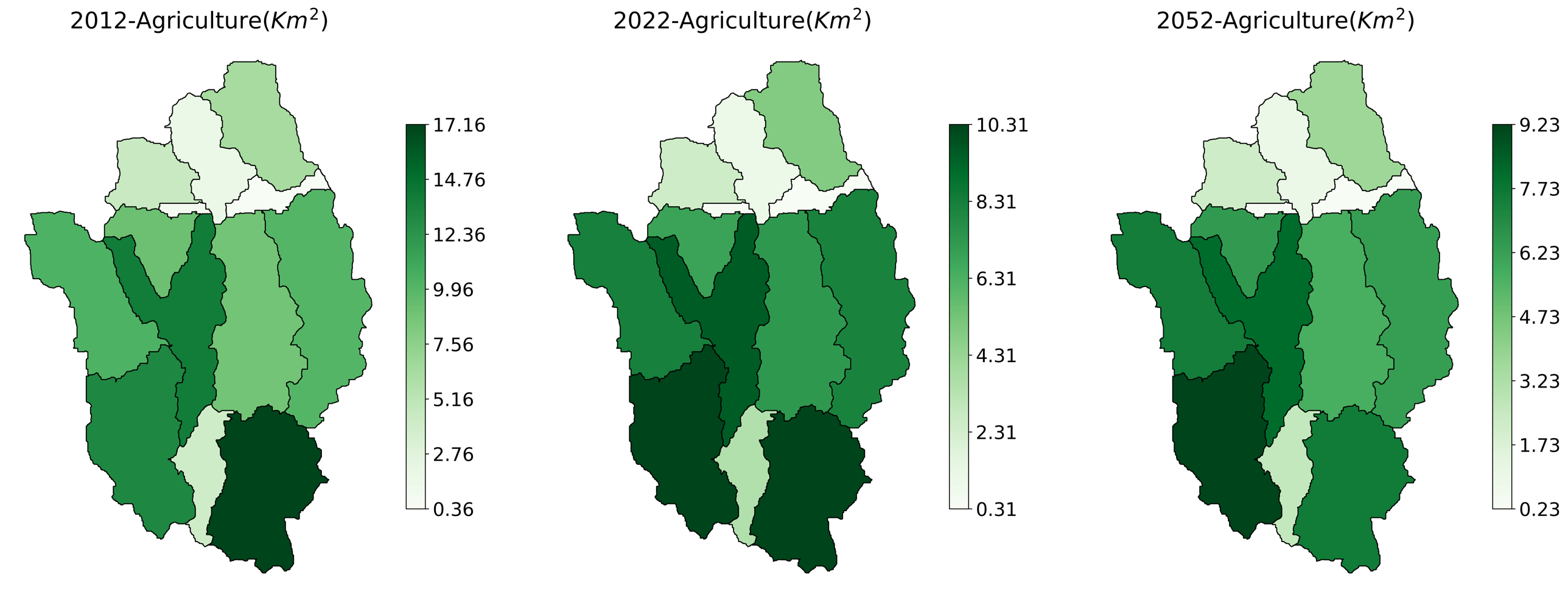


Fig. S3 Agricultural area at each subbasin for 2012, 2022, and 2052


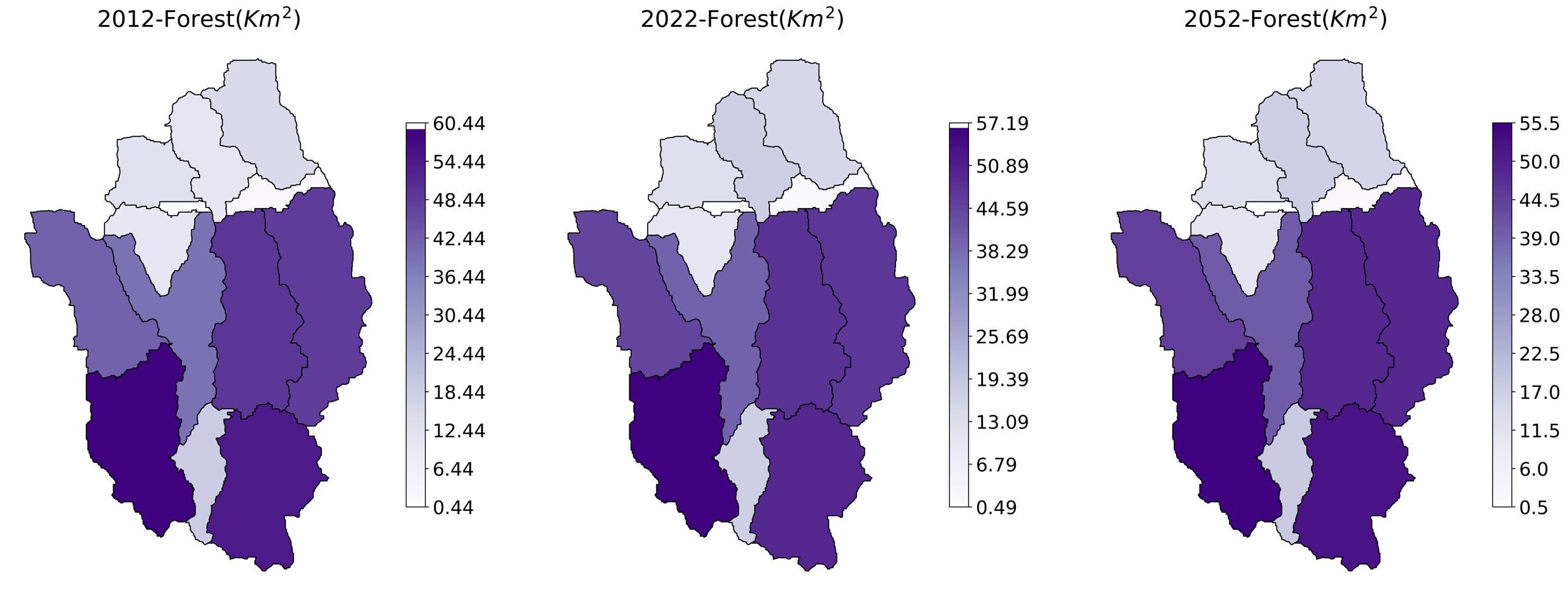


Fig. S4 Forest area at each subbasin for 2012, 2022, and 2052


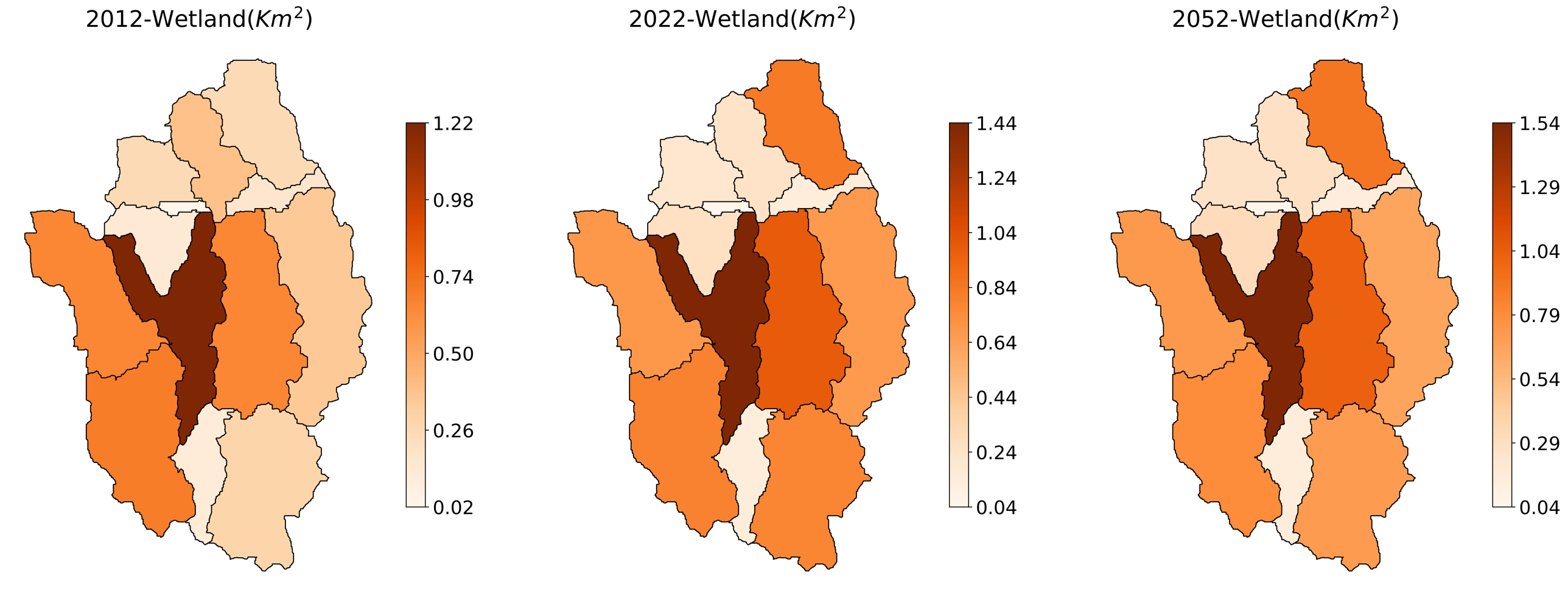


Fig. S5 Wetland area at each subbasin for 2012, 2022, and 2052


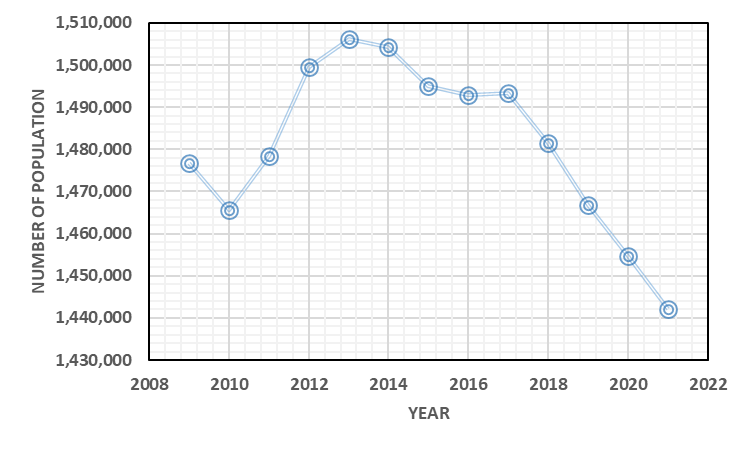


Fig S6. Number of populations in the watershed from 2008 to 2021


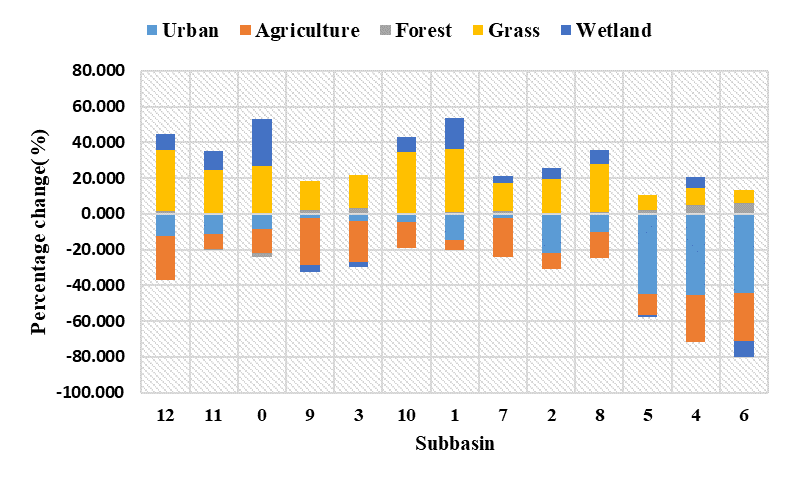


Fig S7. Predicted percentage change for each subbasin for the year 2052


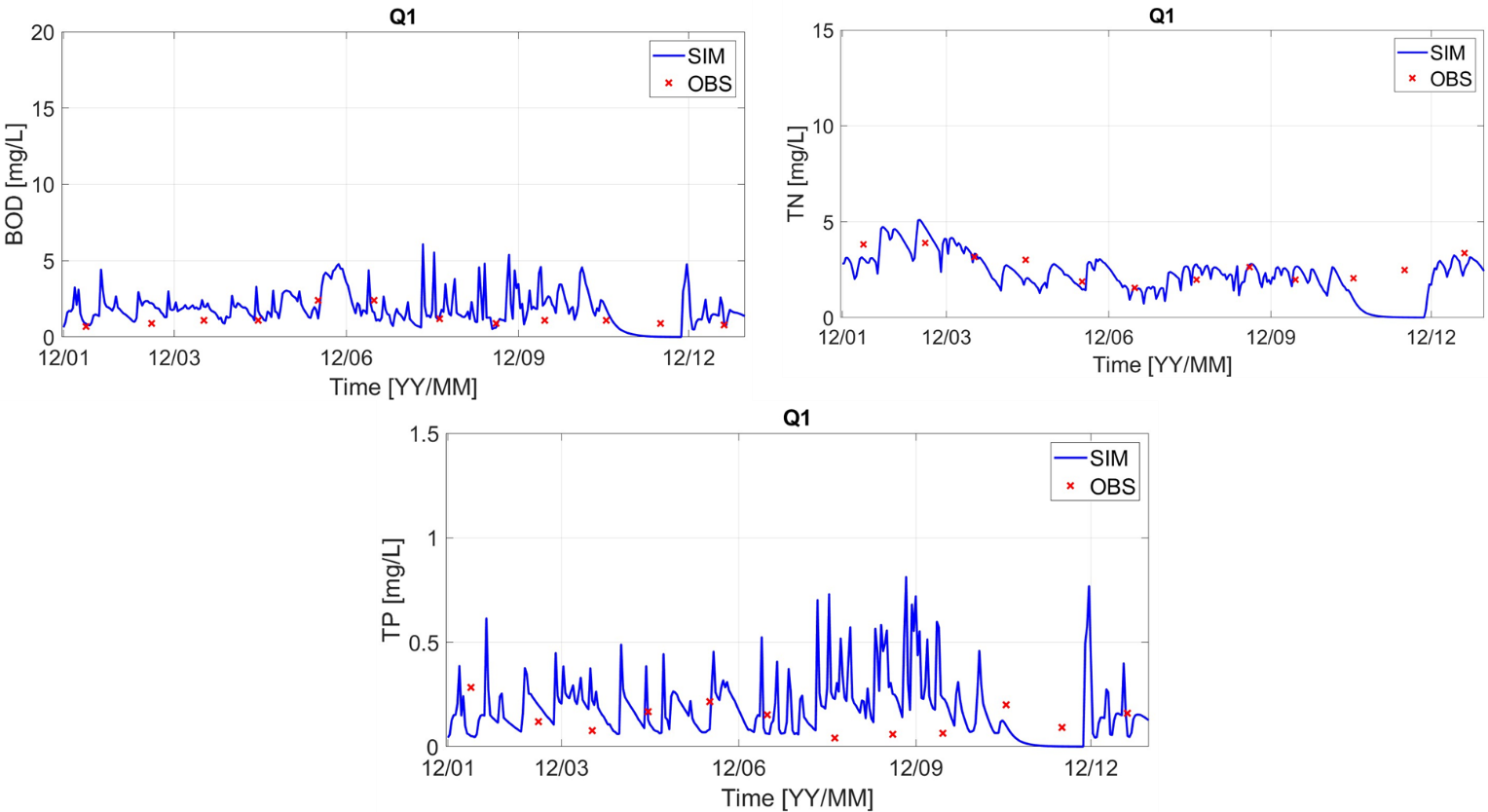


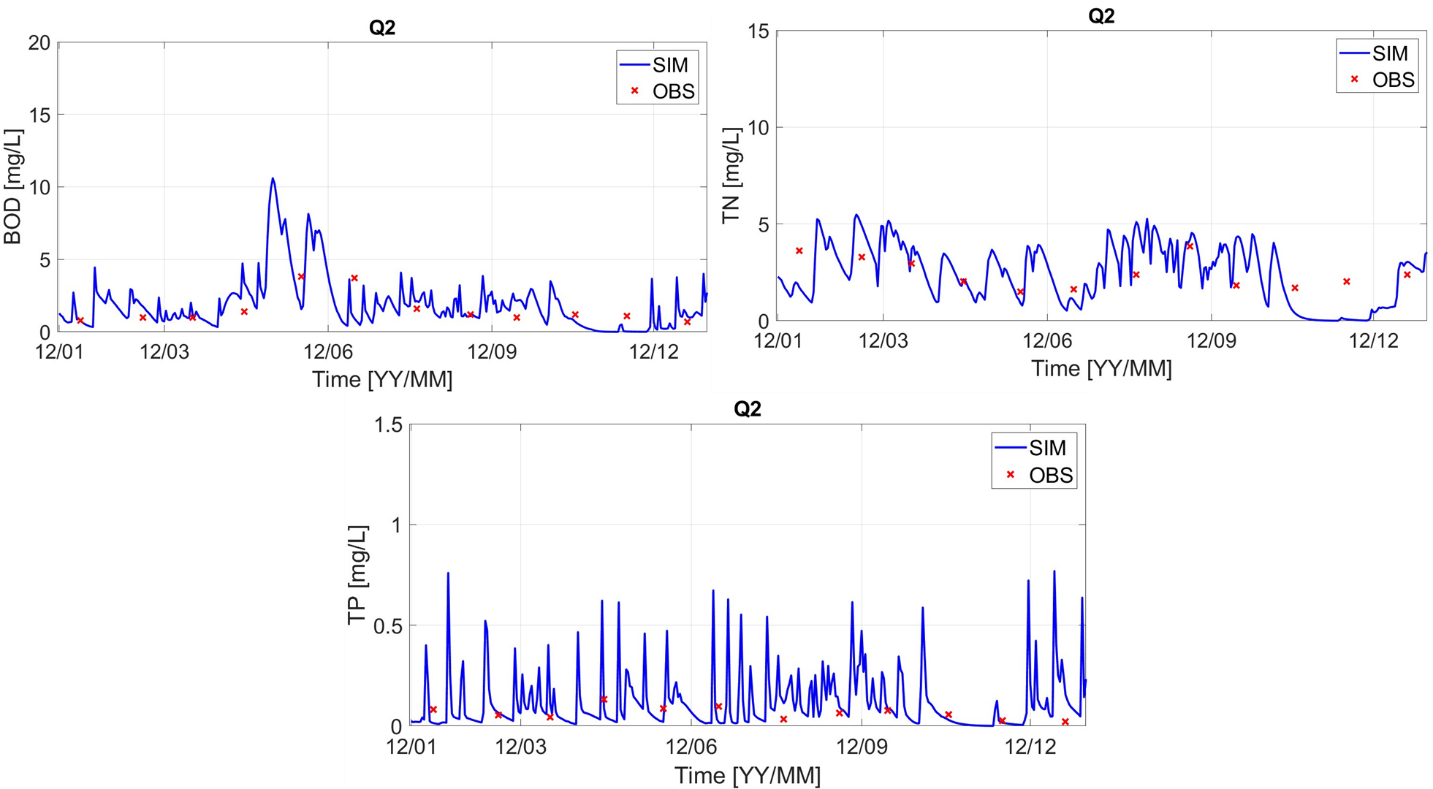


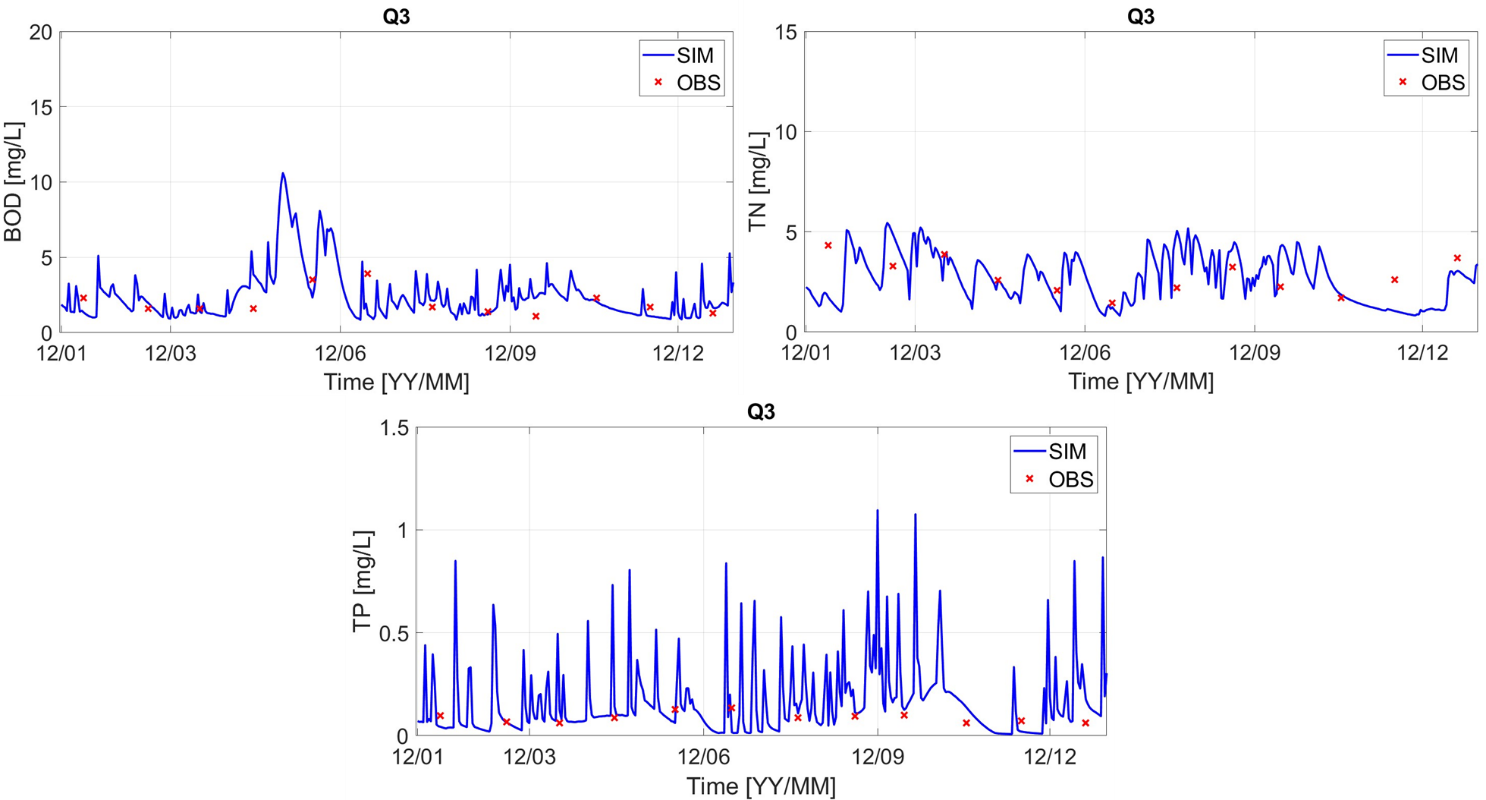


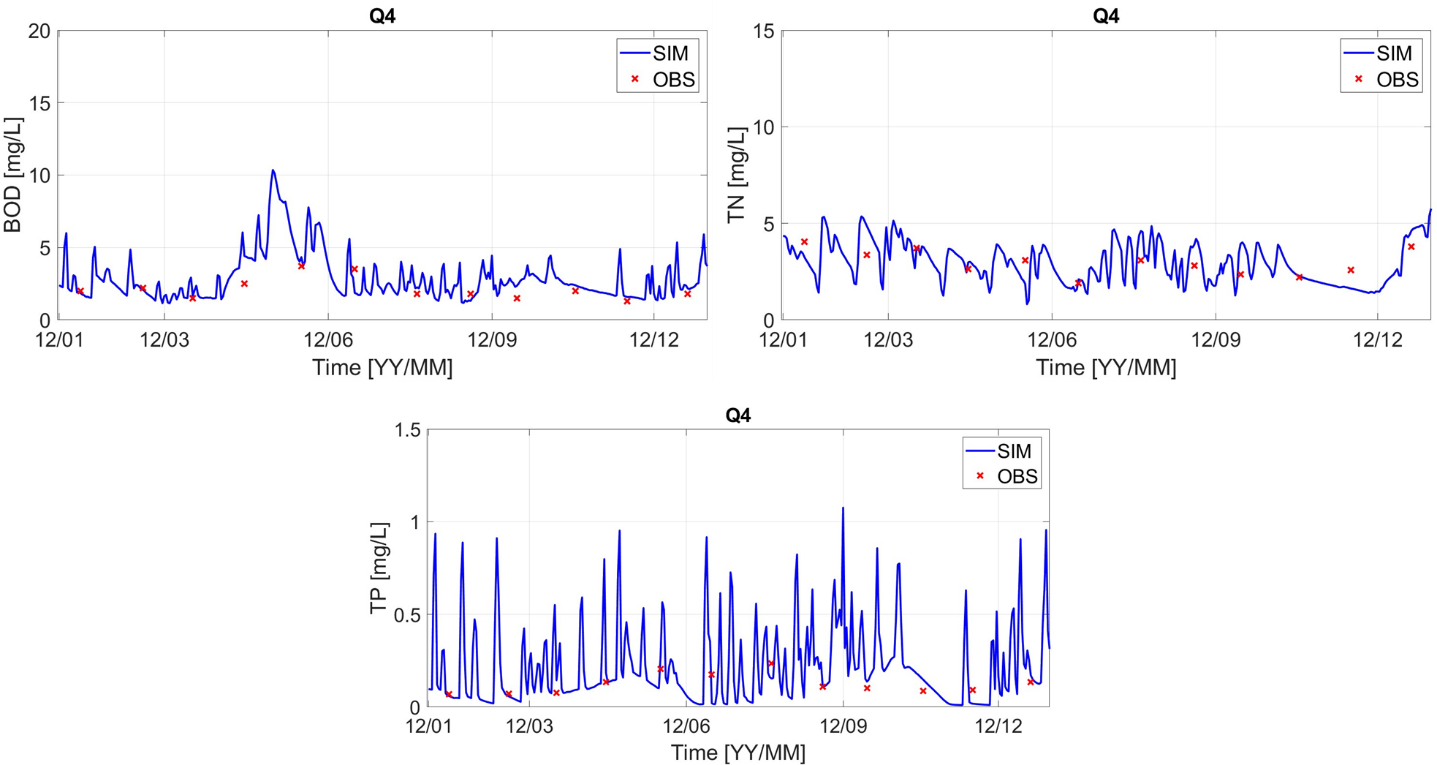


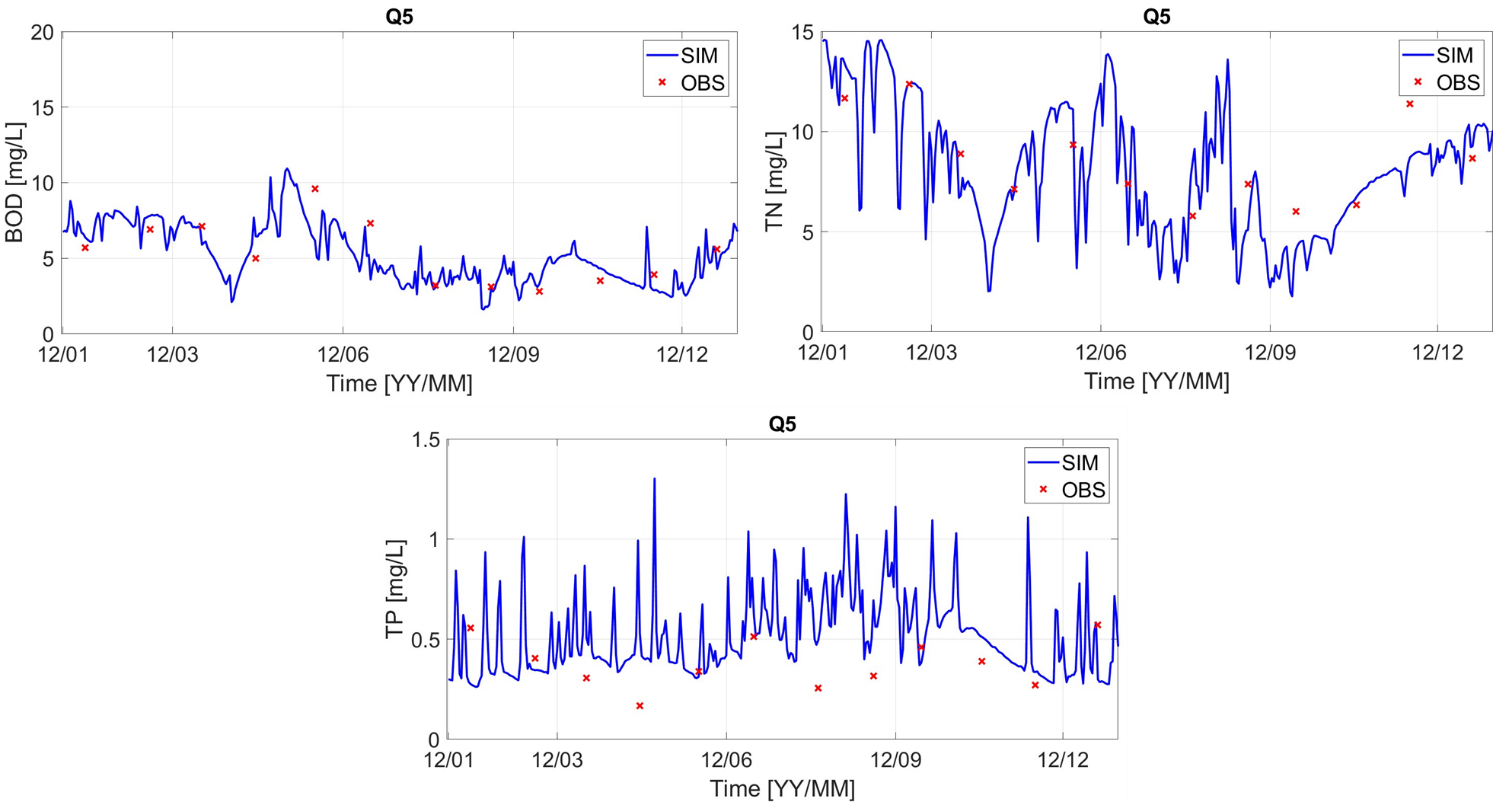


Fig S8. Water quality calibration results for 2012 at Q1-Q5 stations


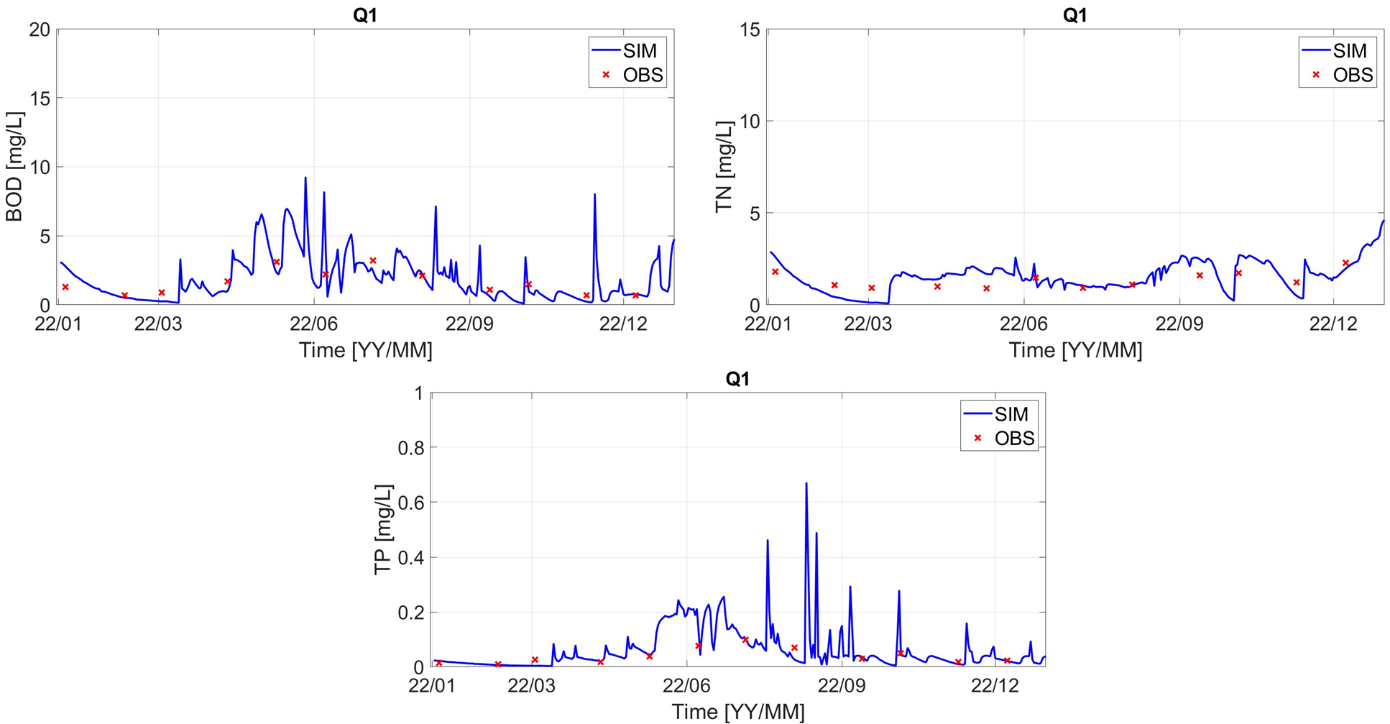


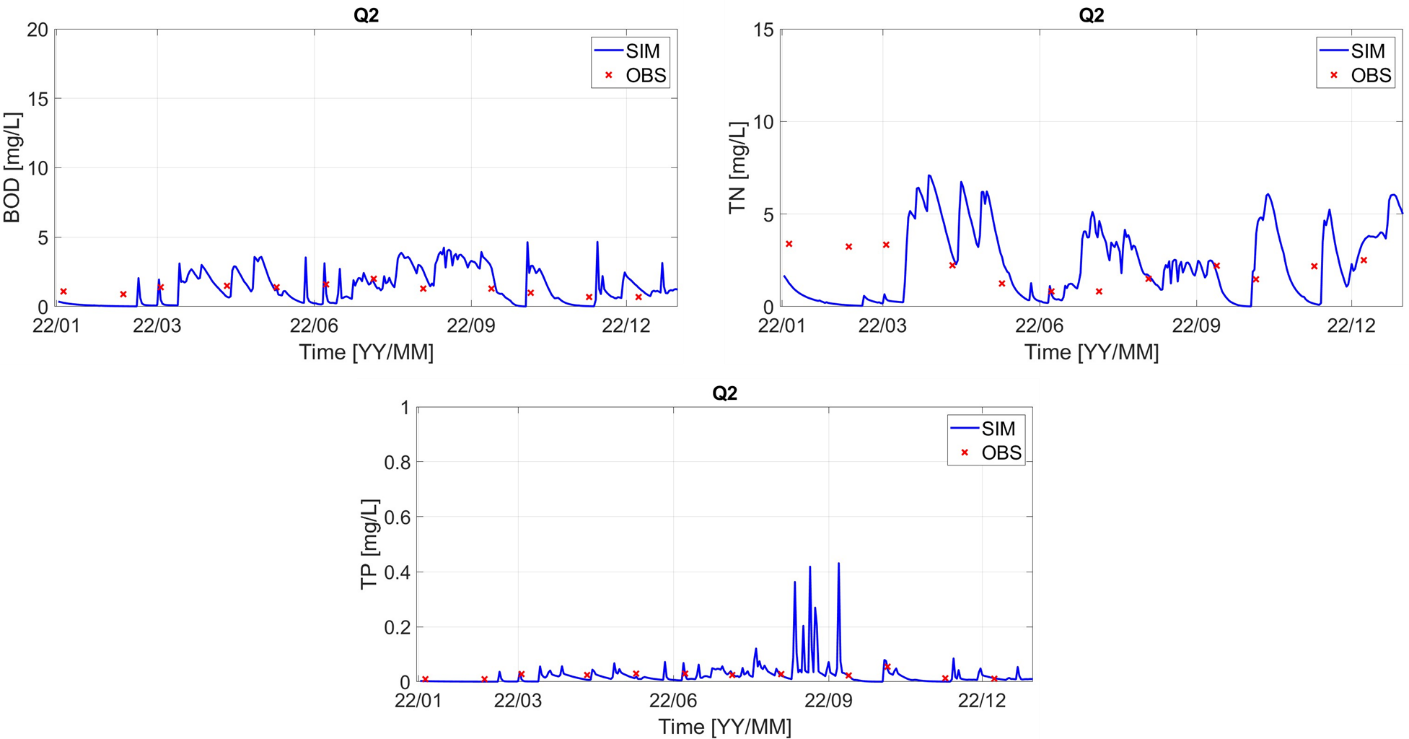


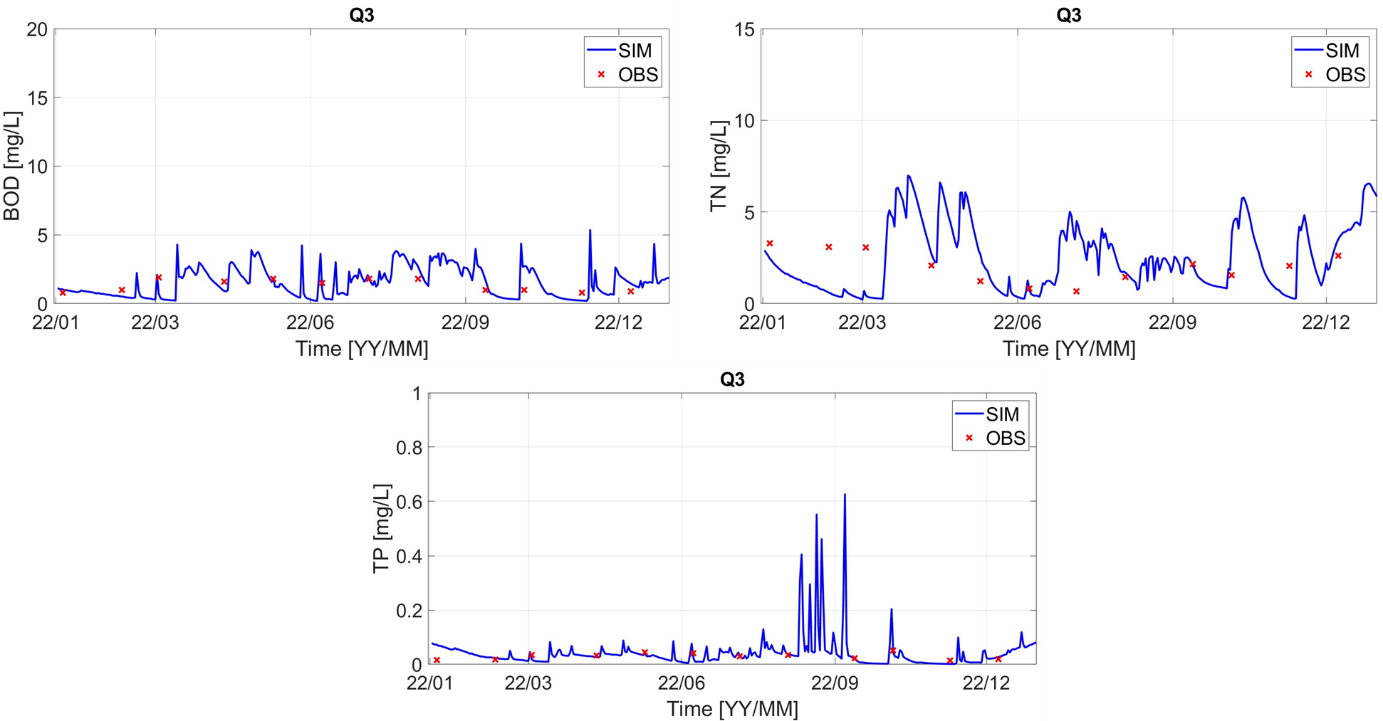


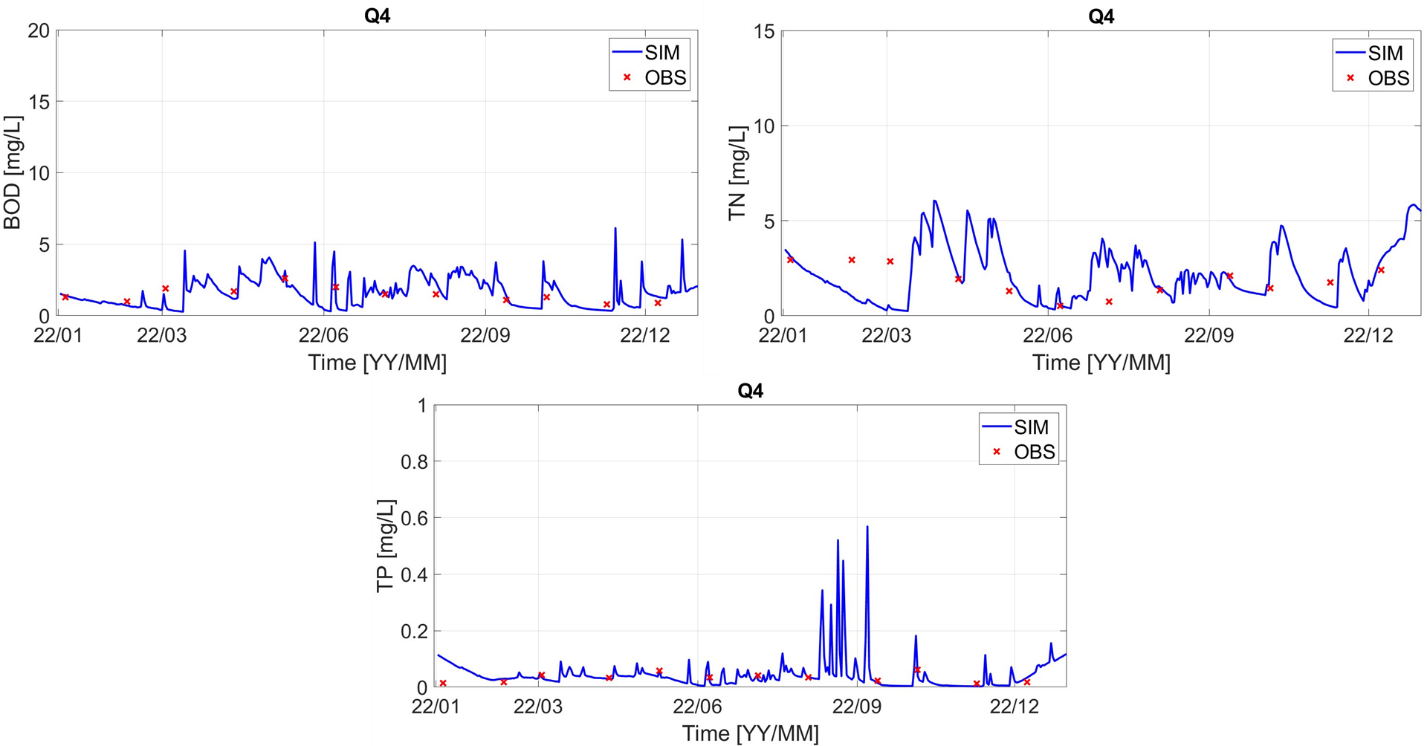


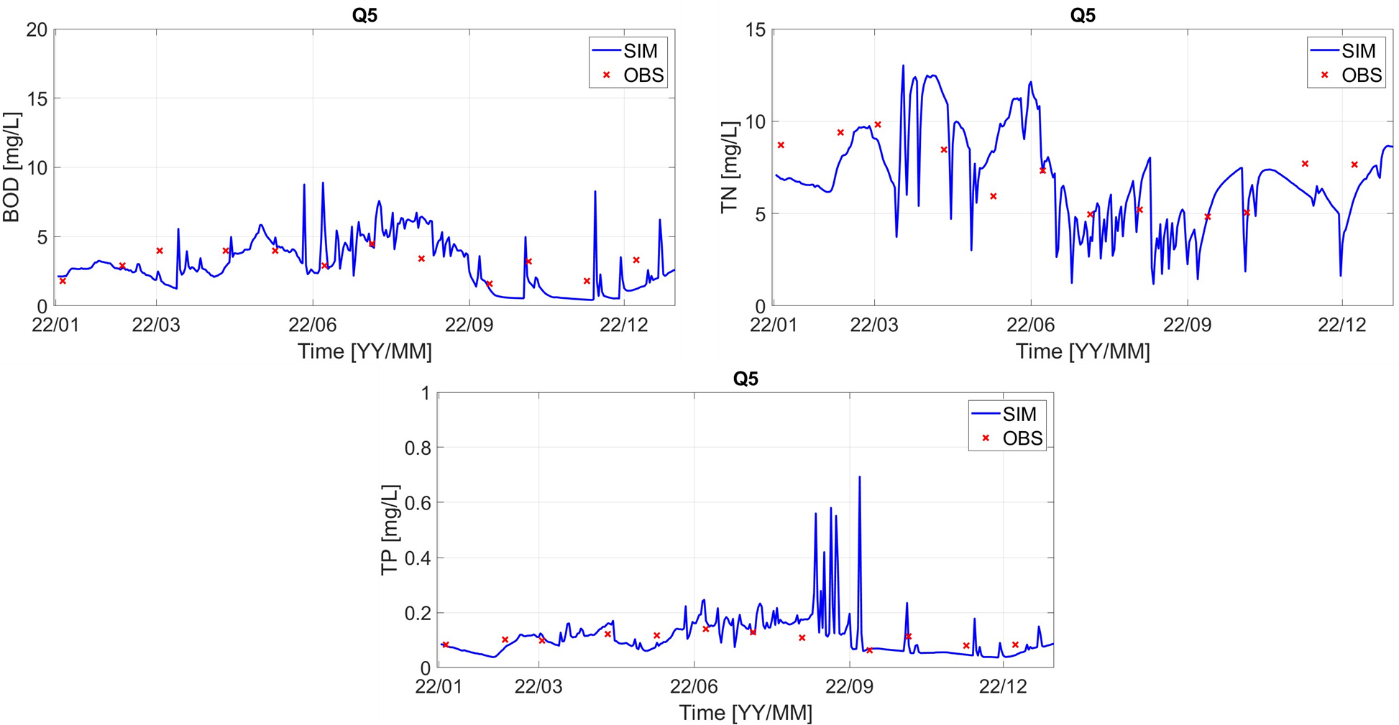


Fig S9. Water quality calibration results for 2022 at Q1-Q5 stations

Table S1. Statistical performance result for water quality in 2010 and 2022.

| **Site** | **WQ variables** | **2012** | | **2022** | |
| --- | --- | --- | --- | --- | --- |
|  |  | PBAIS | MAE | PBAIS | MAE |
| Q1 | BOD | 15.81 | 0.69 | 7.40 | 0.65 |
|  | TN | 6.77 | 0.72 | -7.90 | 0.54 |
|  | TP | 60.80 | 0.10 | 1.13 | 0.01 |
| Q2 | BOD | 16.22 | 0.97 | -1.47 | 0.86 |
|  | TN | -8.86 | 1.26 | 6.0 | 1.68 |
|  | TP | 27.28 | 0.05 | 34.08 | 0.09 |
| Q3 | BOD | 18.25 | 0.98 | -3.68 | 0.64 |
|  | TN | -4.05 | 1.09 | -5.88 | 1.43 |
|  | TP | 2.29 | 0.04 | -2.26 | 0.01 |
| Q4 | BOD | -3.62 | 0.6 | 1.21 | 0.56 |
|  | TN | -10.40 | 0.84 | -4.58 | 1.05 |
|  | TP | -7.75 | 0.04 | -8.37 | 0.01 |
| Q5 | BOD | 14.77 | 1.29 | 3.63 | 1.32 |
|  | TN | 7.95 | 1.60 | 3.93 | 1.30 |
|  | TP | -3.09 | 0.16 | 0.64 | 0.02 |


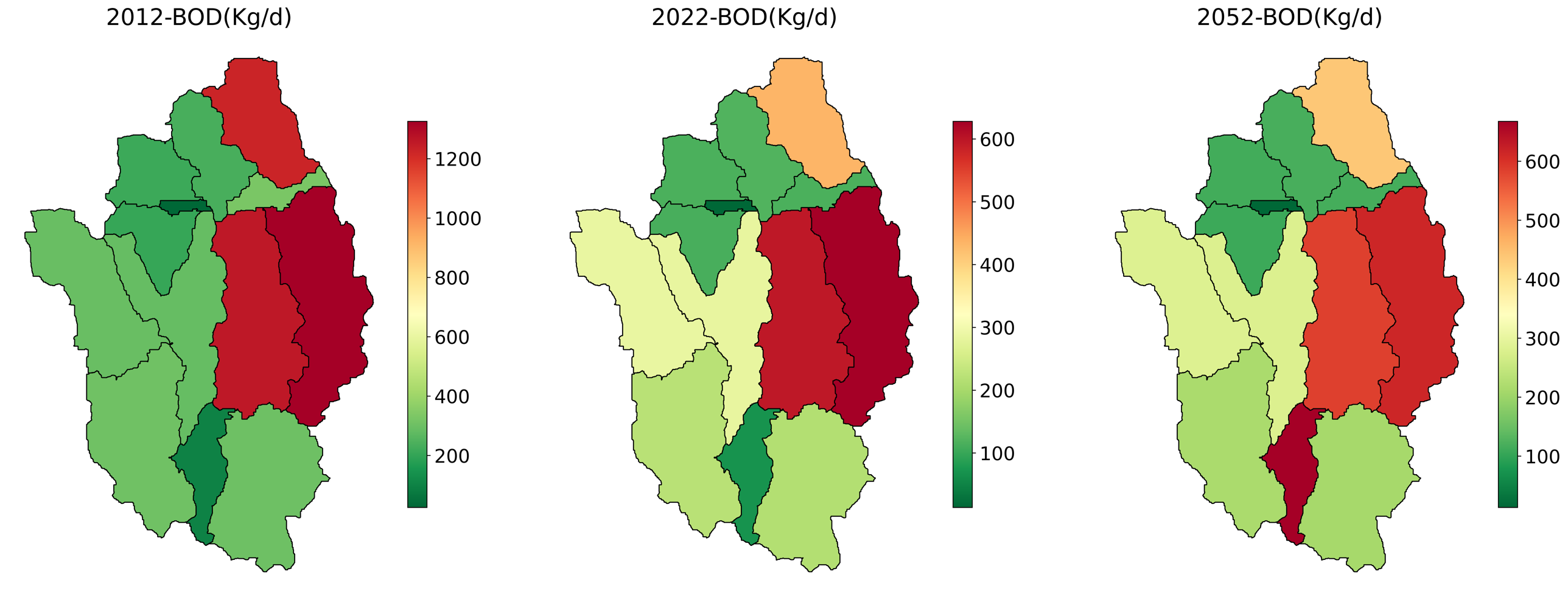


Fig S10. Changes in BOD load at each subbasin for 2012, 2022, and 2052.


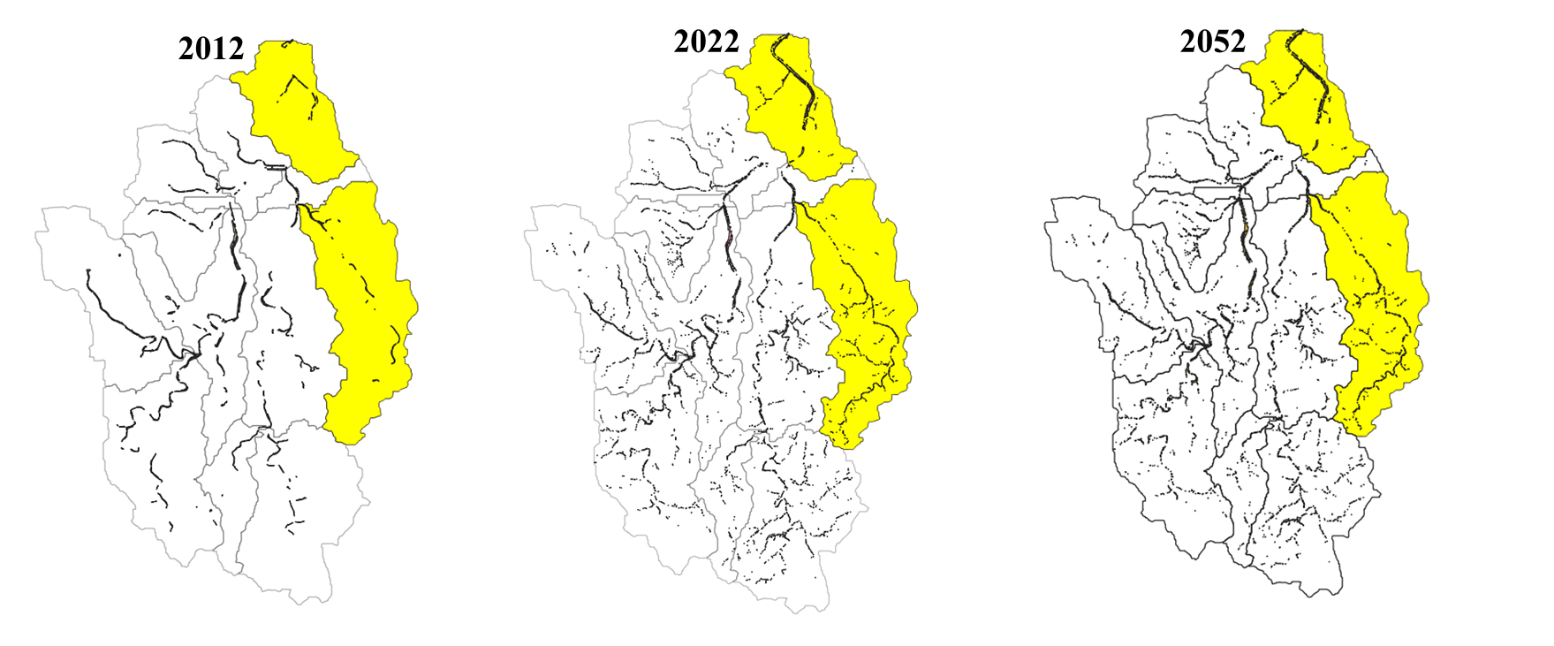


Fig S11. Changes in wetland in subbasin 12 & 3.
